# Protein Profiling of RGS6, a Pleiotropic Gene Implicated in Numerous Neuropsychiatric Disorders, Reveals Multi-Isoformic Expression and a Novel Brain-Specific Isoform

**DOI:** 10.1523/ENEURO.0379-21.2021

**Published:** 2022-01-18

**Authors:** K. E. Ahlers-Dannen, J. Yang, M. M. Spicer, B. Maity, A. Stewart, J. G. Koland, R. A. Fisher

**Affiliations:** Department of Neuroscience and Pharmacology, The Roy J. and Lucille A. Carver College of Medicine, University of Iowa, Iowa City, IA 52242

**Keywords:** expression, isoforms, neuropsychiatric, pleiotropic, RGS6, splicing

## Abstract

A metanalysis identified regulator of G-protein signaling 6 (*RGS6*) as one of 23 loci with pleiotropic effects on four or more human psychiatric disorders. This finding is significant as it confirms/extends the findings of numerous other studies implicating RGS6 in CNS function and pathology. RGS6 is a highly conserved member of the RGS protein family whose cellular roles are likely affected by mRNA splicing and alternative domain inclusion/exclusion. Indeed, we previously identified multiple RGS6 splice variants predicted to produce 36 distinct protein isoforms containing either long (RGS6L) or short (RGS6S) N-terminal domains, an incomplete or intact GGL domain, and nine alternative C termini. Unfortunately, sequence similarities between the isoforms have made it difficult to confirm their individual existence and/or to determine their unique functions. Here, we developed three RGS6-specific antibodies that recognize all RGS6 protein isoforms (RGS6-fl), the N-terminus of RGS6L isoforms (RGS6-L), and an 18-amino acid alternate C-terminal sequence (RGS6-18). Using these antibodies, we demonstrate that RGS6L(+GGL) isoforms, predominating in both mouse (both sexes) CNS and peripheral tissues, are most highly expressed in the CNS. We further identify three novel RGS6 protein bands that are larger (61, 65, and 69-kDa) than the ubiquitously expressed 53- to 57-kDa RGS6L(+GGL) proteins. Importantly, we show that the 69-kDa protein is a brain-specific dephospho form of the 65-kDa band, the first identified phosphorylated RGS6 isoform. Together, these data begin to define the functional significance behind the complexity of *RGS6* gene processing and further clarifies RGS6’s physiological roles by resolving tissue-specific RGS6 protein expression.

## Significance Statement

Psychiatric disorders are highly associated with polygenic variation. Consistent with this, a SNP (single nucleotide polymorphism) (rs2332700) in regulator of G-protein signaling 6 (*RGS6*) is linked to autism spectrum disorder, bipolar disorder, major depression, and schizophrenia. *RGS6* is a highly conserved gene whose complex alternative mRNA splicing produces numerous protein isoforms with high sequence similarity, hindering their functional characterization. Therefore, while aberrant RGS6 signaling and/or expression have been linked to various neuropsychiatric disorders, it is unclear which isoforms are important. This study functionally delineates between the various RGS6 isoforms within mouse. We demonstrate that RGS6 is most highly expressed in CNS, characterize the predominant isoforms, and identify a brain-specific RGS6 protein highly expressed in brain regions associated with various psychiatric disorders.

## Introduction

Psychiatric disorders are a leading cause of worldwide disability and affect >25% of the population (Kessler and Wang, 2008; GBD 2016 Disease and Injury Incidence and Prevalence Collaborators, 2017). Research in this area has noted that risk is highly associated with polygenic variation (Sullivan et al., 2018; Smoller et al., 2019). Furthermore, it is now clear that psychiatric disorders are not distinct and there is significant genetic overlap between them (Cross-Disorder Group of the Psychiatric Genomics Consortium, 2013; Anttila et al., 2018). Highlighting this point, a recent meta-analysis conducted by the Cross-Disorder Group of the Psychiatric Genomic Consortium (Cross-Disorder Group of the Psychiatric Genomics Consortium, 2019) of 232,964 psychiatric patients and 494,162 controls revealed 23 genetic loci with significant pleiotropic effects on ≥4/8 disorders studied: anorexia nervosa, attention deficit hyperactivity disorder, autism spectrum disorder, bipolar disorder, major depression, obsessive-compulsive disorder, schizophrenia, and Tourette syndrome.

**Figure 1.**
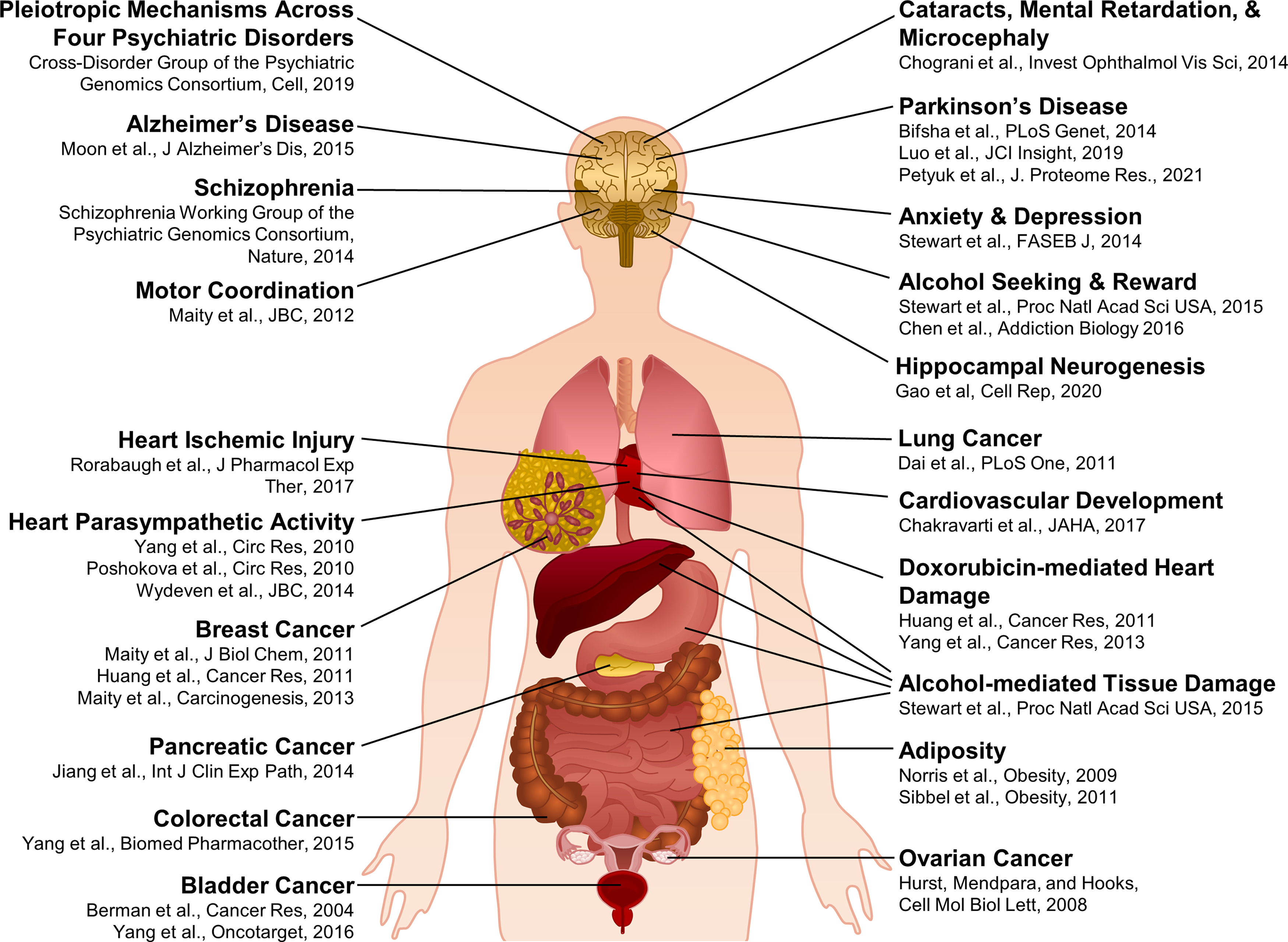
RGS6 has numerous neuropsychiatric, physiological, and pathophysiological roles. This diagram highlights the many neuropsychiatric, physiological, and pathophysiological functions that have been attributed to RGS6 as well as the primary literature describing these functions.

One of the 23 pleiotropic genetic loci described was a SNP (rs2332700) in regulator of G-protein signaling 6 (*RGS6*), previously identified in a GWAS of schizophrenic patients (Schizophrenia Working Group of the Psychiatric Genomics Consortium, 2014), that was linked not only to schizophrenia, but also to autism spectrum disorder, bipolar disorder, and major depression. This finding is significant because of the study’s magnitude and as it confirms/extends the findings of other human and animal studies implicating RGS6 in CNS function and pathology (Fig. 1). In particular, alterations in RGS6 signaling and/or expression have been associated with: alcohol use disorders (rs11621871; Stewart et al., 2015; Chen et al., 2017), anxiety/depression (Stewart et al., 2014), Parkinson’s disease (Bifsha et al., 2014; Luo et al., 2019; Petyuk et al., 2021), Alzheimer’s disease (rs4899412; Moon et al., 2015), motor coordination (Maity et al., 2012), adult hippocampal neurogenesis (Gao et al., 2020), as well as human cataracts, mental retardation, and microcephaly (c. 1369–1 G > C; Chograni et al., 2014).

**Figure 2.**
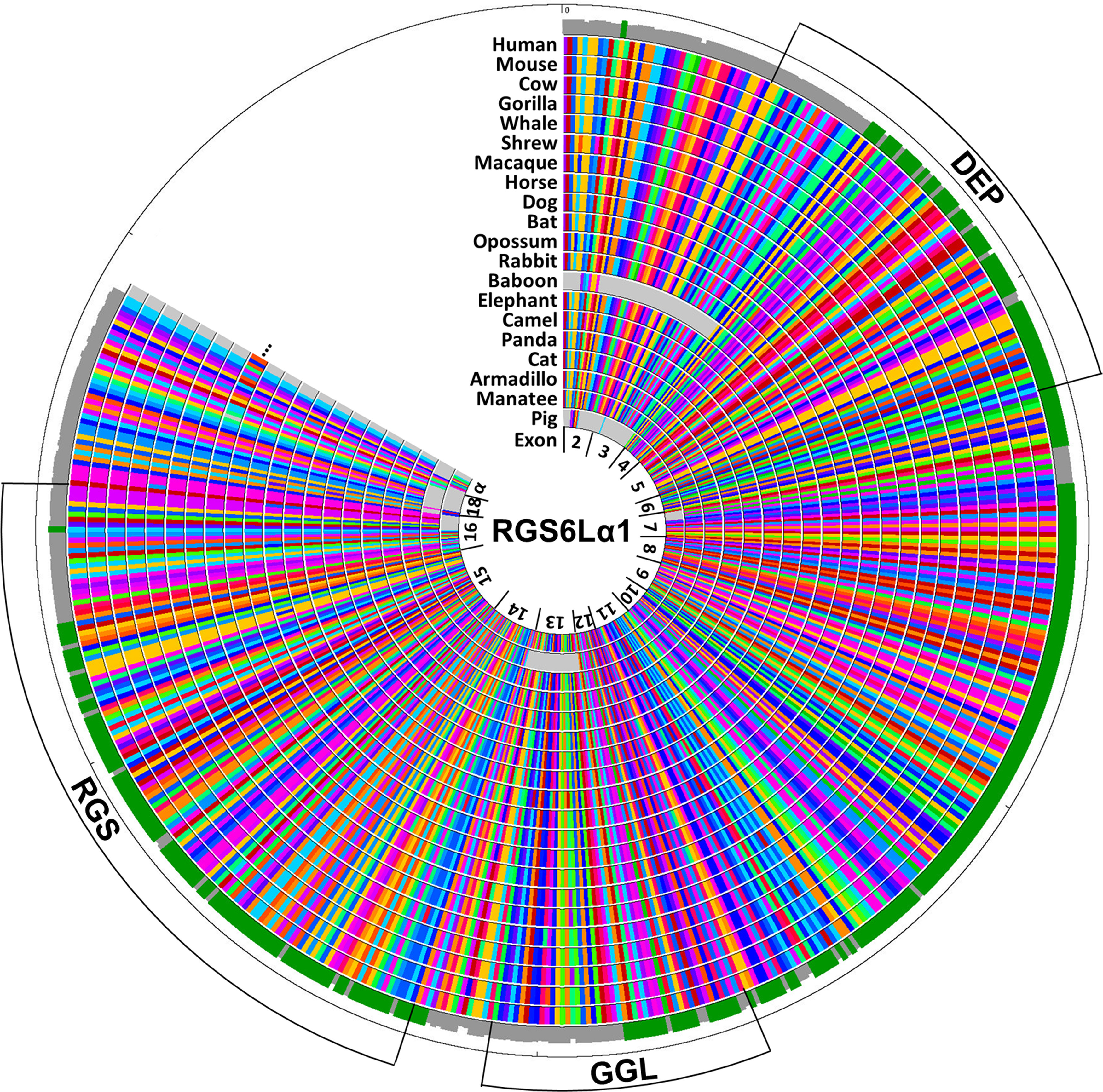
The RGS6 protein sequence is highly conserved across the animal kingdom. This figure shows a conservation map of the largest RGS6 isoform we previously identified (Chatterjee et al., 2003) in different mammalian species. This isoform is designated RGS6Lα1(+GGL) as it includes the extended N-terminus found in RGS6 long (RGS6L) isoforms, the GGL domain, as well as the alternative α1 C-terminal sequence encoded by exons 18 and α. Each column within the conservation map represents a single amino acid and conservation of color within a column represents amino acid conservation. When looking at the histogram around the outside of the conservation map, green represents 100% amino acid conservation whereas gray represents <100% conservation.

**Figure 3.**
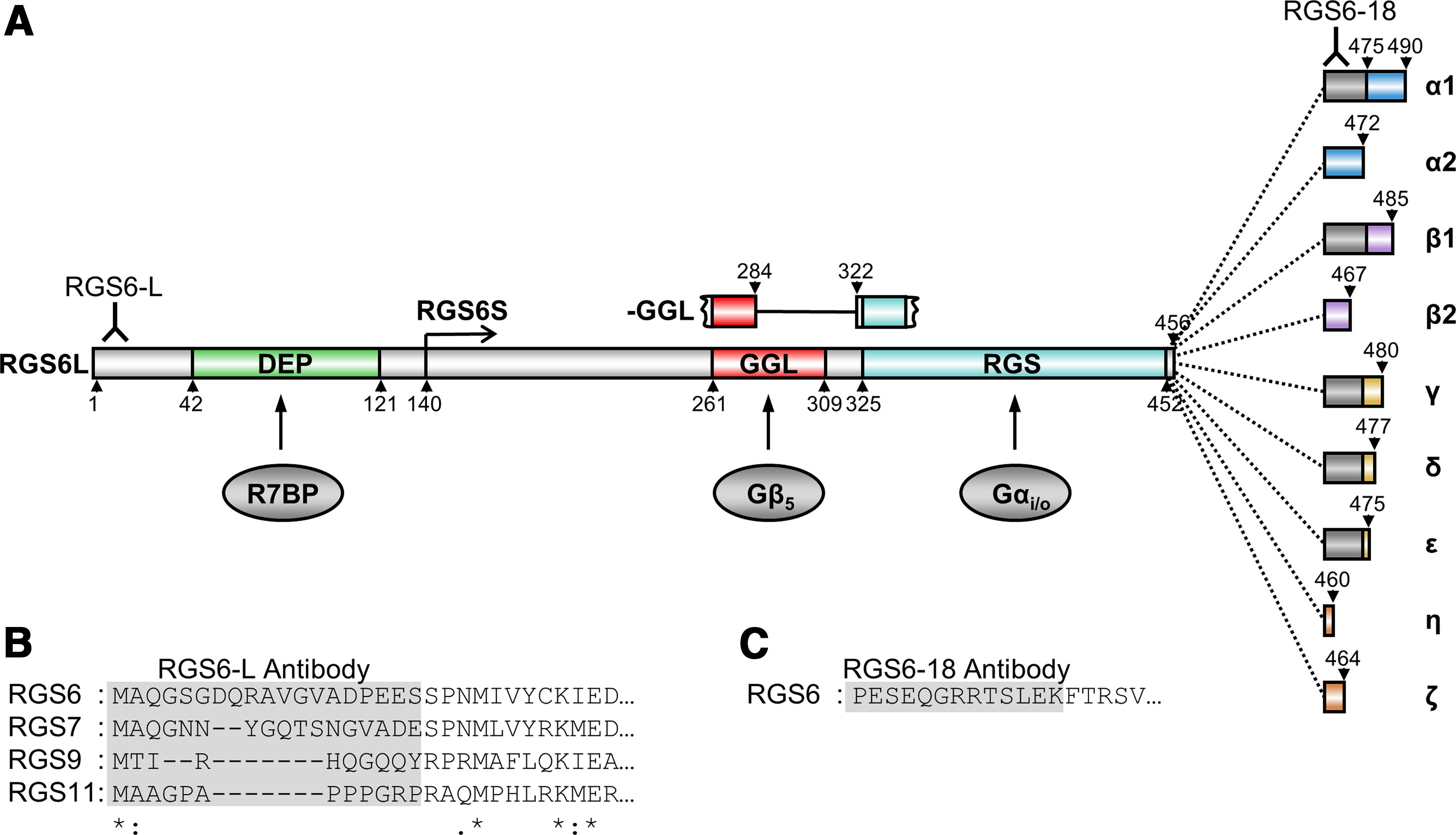
Predicted protein structure and binding partners for human RGS6 protein isoforms. RGS6 is a member of the R7 subfamily (RGSs 6, 7, 9, 11) distinguished by two additional domains, DEP and GGL, which target these proteins to the membrane (through interaction with R9AP and/or R7BP) and promote their stability (through interaction with Gβ_5_), respectively. In addition to DEP-mediated and GGL-mediated spatial function and regulation, RGS6’s cellular roles are also likely affected by mRNA splicing and alternative domain inclusion/exclusion. ***A***, Illustration of the 36 RGS6 protein isoforms encoded by RGS6 mRNA splice variants we identified (Chatterjee et al., 2003). RGS6 proteins possess either long (RGS6L) or short (RGS6S) N-terminal domains, an incomplete or intact GGL domain, and nine alternative C-terminal sequences. ***B***, Sequence alignment of the N-terminal region of RGS6 to that of RGS7, RGS9, and RGS11. The gray box represents a region of low sequence conservation that was used to develop the RGS6L antibody, RGS6-L. ***C***, The protein sequence of an alternatively spliced region of RGS6 present in the C terminus of 56% of RGS6 protein isoforms that was used to develop RGS6-18 antibody. The approximate location at which the RGS6-L and RGS6-18 antibodies bind to RGS6 proteins is denoted in ***A*** by an antibody icon.

RGS proteins modulate the magnitude and duration of G protein-coupled receptor (GPCR) signaling by facilitating heterotrimeric G-protein inactivation, a function bestowed by their RGS domain. RGS6 is a highly conserved (Fig. 2) member of the R7 subfamily, which modulates Gα_i/o_ signaling and is distinguished by two additional domains, DEP and GGL, responsible for both membrane targeting and protein stability (Ross and Wilkie, 2000; Hooks et al., 2003). RGS6’s cellular roles may also be affected by mRNA splicing and alternative domain inclusion/exclusion. Indeed, multiple RGS6 splice variants have been identified which are predicted to produce 36 distinct RGS6 protein isoforms containing either long (RGS6L) or short (RGS6S) N-terminal domains, an incomplete or intact GGL domain (−/+GGL), and nine alternative C-termini (Fig. 3; Chatterjee et al., 2003). Unfortunately, because of sequence similarity it has been difficult to confirm the existence of many of these isoforms or to determine their function. Therefore, while aberrant RGS6 signaling and/or expression have been linked to various neuropsychiatric disorders, it is unclear which RGS6 isoforms are important.

Adding further complexity are studies demonstrating RGS6 has critical physiological and pathophysiological functions outside the CNS as well. In the periphery RGS6 has been shown to: act as a critical tumor suppressor (Berman et al., 2004; Huang et al., 2011; Dai et al., 2011; Maity et al., 2011, 2013; Jiang et al., 2014; Luo et al., 2015; Yang et al., 2016), negatively modulate parasympathetic heart regulation (Posokhova et al., 2010; Yang et al., 2010; Wydeven et al., 2014), promote cardiovascular development (Chakravarti et al., 2017), associate with adiposity (Sibbel et al., 2011), and mediate heart-ischemic injury (Rorabaugh et al., 2017), doxorubicin-induced cardiac toxicity (Huang et al., 2011; Yang et al., 2013), as well as alcohol-induced cardiac, hepatic, and gastrointestinal damage (Stewart et al., 2015; Fig. 1). Interestingly, not all of these peripheral functions (i.e., tumor suppression) rely on RGS6’s ability to negatively regulate Gα_i/o_ (Maity et al., 2011).

As the list of RGS6’s critical physiological and pathophysiological roles continues to grow, particularly with regards to neuropsychiatric disorders, it is imperative to perform a comprehensive analysis of RGS6 isoform expression. We hypothesize such an analysis will help to clarify the functional significance behind the complexity of *RGS6* gene processing.

## Materials and Methods

### Mice

We have previously described the generation of and genotyping methods for RGS6^−/−^ mice (Yang et al., 2010). For these studies, RGS6^−/−^ mice were back-crossed onto the C57BL6 background for five generations. Three-month-old male and female RGS6^+/+^ and RGS6^−/−^ mice were used for the experiments described in the manuscript. All animal procedures described were approved by the University of Iowa Institute for Animal Care and Use Committee.

### RGS6Lα1(+GGL) conservation map

NCBI’s predicted protein sequence for human RGS6Lα1 (+GGL) (isoform 1-NP_001191245.1), the largest RGS6 isoform identified by Chatterjee et al. (2003) was BLASTed against the following taxids: *Gorilla*, *Macaca*, *Papio*, *Soricidae*, *Mus*, *Oryctolagus*, *Sus*, *Camelus*, *Delphinus*, *Orcinus*, *Bos bovis*, *Ovis*, *Equus*, *Felis*, *Canis canis*, *Ailuropoda*, *Vulpes*, *Chiroptera*, *Loxodonta*, *Trichechus*, *Dasypodidae*, and *Didelphimorphia* using NCBIs default settings. The sequence IDs with the highest amino acid conservation were placed in the MegAlign program (part of the DNASTAR Lasergene 12 Core Suit) and aligned using the Clustal W Method. This alignment was used to generate a circular map using the DNASTAR GenVision program.

### RGS6 structure homology modeling

A structural model of RGS6Lα1(+GGL) based on the RGS9/Gβ_5_ complex structure (2PBI; Cheever et al., 2008) was created using the Swiss-PdbViewer (Swiss Institute of Bioinformatics). Domains within the predicted RGS6Lα1 (+GGL) amino acid sequence that did not align with the RGS9/Gβ_5_ complex were modeled with an in-house algorithm (clean.swiss. pl) created by the Elcock lab (University of Iowa), which implements the Loopy program (Xiang et al., 2002). The model was visualized with VMD (Theoretical and Computational Biophysics Group, Beckman Institute, University of Illinois at Urbana-Champaign).

### Cell culture and transfection for protein half-life assays

HEK293T cells were plated in 12-well plates at 3.5 × 10^5^ cells/well and were cultured in high-glucose DMEM (Sigma-Aldrich) supplemented with 10% fetal bovine serum (Invitrogen 26140), 100 units/ml penicillin (Invitrogen 15140), and 100 μg/ml streptomycin (Invitrogen 15140); 24 h later, cells were transiently co-transfected using the Lipofectamine 2000 system (ThermoFisher Scientific 116680) with a pcDNA3.1-HA vector containing Gβ_5_ (Gβ_5_-HA; gifted by Songhai Chen), a pEGFP vector containing R7BP (R7BP-GFP; gifted by Martemyanov, Scripps, FL), and a pcDNA3.1 vector containing RGS6 (1:1:2 ratio, total DNA = 1 μg/well). Transfection media was exchanged for new supplemented DMEM media at 6 h following transfection; 24 h posttransfection, supplemented DMEM was removed, and cells were cultured in serum free supplemented DMEM containing 100 μm cycloheximide (Sigma C6255) for 0, 2, 4, 6, 12, and 24 h.

### Antibody generation

Three different polyclonal RGS6 antibodies were used in this study. Antibodies to the N-terminal domain of RGS6L (RGS6-L) were generated with a synthetic peptide immunogen corresponding to residues 1–19 (MAQGSGDQRAVGVADPEESC-COOH) of RGS6L (Biosynthesis Inc; Chatterjee et al., 2003). Antibodies to the unique splice forms of RGS6 that retain exon 18 sequences (RGS6-18) were generated (Biosynthesis Inc.) with a peptide immunogen corresponding to 14 amino acids in this region (–CKPESEQGRRTSLEK; Chatterjee et al., 2003). Antibodies to full length RGS6L (RGS6-fl) were generated (Elmira Biologicals) to recombinant RGS6L following its expression, solubilization, and purification from inclusion bodies in bacteria.

### Western blotting

#### Cell culture

Media was aspirated off cells and 100 μl of ice cold RIPA buffer (150 mm NaCl, 1% NP-40, 0.5% sodium deoxycholate, 0.1% sodium dodecyl sulfate, and 50 mm Tris-HCl; pH 8) containing protease (Roche 11836170001) and phosphatase (Sigma p5726) inhibitors was added to each well. Cells and RIPA were then placed in prechilled tubes, vortexed for 30 s, incubated on ice for 5 min, and centrifuged at 8000 rpm (4°C) for 10 min. Supernatant was transferred to tubes containing 4× SDS PAGE sample buffer and boiled for 5 min before loading onto gels.

#### Tissues

Tissues were prepared for Western blot analysis as previously described (Yang et al., 2016).

Western blots were probed with home-made rabbit anti-RGS6-specific antibodies (RGS6-L, RGS6-18, and RGS6-fl; 1:1000–2000), rabbit anti-Gβ5 (a gift from William F Simonds, 1:1000 dilution; Zhang et al., 1996), rabbit anti-RGS7 (a gift from Vladlen Z. Slepak, 1:1000 dilution; Levay et al., 1999), rabbit anti-RGS9-2 (a gift from Steve Gold, 1:1000 dilution; Psifogeorgou et al., 2007; Psigfogeorgou et al., 2011), mouse-anti-RGS11 (a gift from Jason Chen, 1:1000 dilution; Chen et al., 2010), as well as commercial mouse monoclonal anti-α-tubulin (Calbiochem, CP06-100UG, 1:5000 dilution) and rabbit anti-actin (Sigma A2066, 1:1000), primary antibodies. LI-COR Odyssey goat anti-mouse (926-32220 and 926-32210) and goat anti-rabbit (926-32211 and 926-68071) secondary antibodies were used at a 1:10,000 dilution for protein visualization with the Odyssey system.

### Immunohistochemistry

Mice were perfused with a saline solution at a rate of 1 ml/min for 5 min, followed by infusion of a freshly made fixative solution (4% paraformaldehyde, 23 mM NaH_2_PO_4_, and 77 mM Na_2_HPO_4_) at a rate of 1 ml/min for 15 min. Tissues were collected and soaked in the fixative solution (4% paraformaldehyde, 23 mM NaH_2_PO_4_, and 77 mM Na_2_HPO_4_) overnight. After fixation, tissues were embedded in paraffin and sectioned. Tissue sections of RGS6^+/+^ and RGS6^−/−^ mice were processed to examine expression of RGS6 as we described previously (Maity et al., 2011). Briefly, sections were dewaxed in xylene, treated with a graded series of alcohol solutions, immersed in 3% hydrogen peroxide to block endogenous peroxide activity, blocked with 5% bovine serum albumin and then incubated overnight at 4°C with rabbit anti-RGS6-fl antibody. Following washing (3 × 10 min) in PBS, sections were incubated for 1 h at room temperature with peroxidase-conjugated secondary antibodies (Cell Signaling, 7074). A positive reaction was detected by exposure to stable diamin-obenzidene (General Biosciences Corporation) for 3 min. The sections were counterstained in Harris hematoxylin and observed under the microscope. This is true for all tissues samples except heart. For mice hearts, frozen sections were blocked for 1 h at 4°C in blocking buffer, containing 10% goat serum, 0.3% Triton X-100, 80.4 mM Na_2_HPO_4_, 19.0 mM NaH_2_PO_4_, pH7.4, and then incubated overnight at 4°C with rabbit anti-RGS6-fl antibody. Following washing (3 × 10 min) in the blocking butter, sections were incubated for 1 h at room temperature with Alexa-conjugated secondary antibodies (Fisher Scientific, A11011).

### Bioinformatic prediction of RGS6 phosphorylation sites

Putative phosphorylation sites in RGS6Lα1(+GGL) were identified using the Group-based Prediction System (GPS) 3.0 from Cuckoo Workgroup. This software uses hierarchical grouping of protein kinases to identify possible kinase-specific or family-specific phosphorylation sites in each protein sequence based on a metric measuring similarity of the protein sequence to known phosphorylation site motifs. The software considers each specific serine/threonine/tyrosine residue and 7 amino acids up and downstream of that residue in generating a prediction score, a measure of the likelihood of peptide phosphorylation. In our analysis, the threshold was set to high and predicted site/kinases were considered if their GPS prediction score was at least 1 above the cutoff value defined by the software (ΔScore ≥ 1). Phosphorylation sites and corresponding kinases meeting these criteria are listed in Extended Data Figure 7-1. Subsequently, a diagram depicting each putative RGS6 phosphorylation site and the corresponding kinase with the highest prediction score was constructed (Fig. 7*A*).

### Phosphatase/kinase assays

Analysis of RGS6 phosphorylation and de-phosphorylation was assayed in RGS6^+/+^ and RGS6^−/−^ whole-brain tissue lysates. Tissues were homogenized in the absence of phosphatase inhibitor cocktail. Lysates were diluted to a final concentration of 2 μg/μl in 1× NEBuffer Pack for Protein MetalloPhosphatases (New England BioLabs). A total of 100 units λ phosphatase (New England BioLabs) were added to each reaction as indicated. For kinase assays, lysates were supplemented with 2 mM ATP and 1 mM MgCl_2_ required for enzyme activity, and with or without activators of PKA, PKC, or Ca^2+^-stimulated kinases as shown in Figure 7*D*; 30 mM NaF was added to reactions lacking λ phosphatase and in kinase reactions to inhibit endogenous phosphatases. Reactions were incubated at 30°C for 20 min and terminated through the addition of 1× sample buffer. Samples were subjected to SDS-PAGE and immunoblotting as described above.

### Experimental design and statistical analysis

All graphical data are expressed as mean ± SEM. All sample sizes reported are based on conservative power analyses estimating β = 0.2 (80%) with statistical significance being evaluated against α = 0.05. Effect sizes were estimated using Cohen’s *d* formulation before calculating power analyses. Significant differences within tissue expression data (Figs. 5*B*, 8*A*, 9*A*; Extended Data Figs. 5-1, 8-1, and 9-1) were analyzed via two-way ANOVA with Fisher’s LSD *post hoc* adjustment. Significant differences within immunoreactivity data (Fig. 7*C*,*E*) were also analyzed via two-way ANOVA with Fisher’s LSD *post hoc* adjustment. All data were analyzed using XLSTAT software.

## Results

### Development and characterization of RGS6 antibodies confirms *in vivo* expression of RGS6L(+GGL) and identifies novel RGS6 protein bands

**Figure 4.**
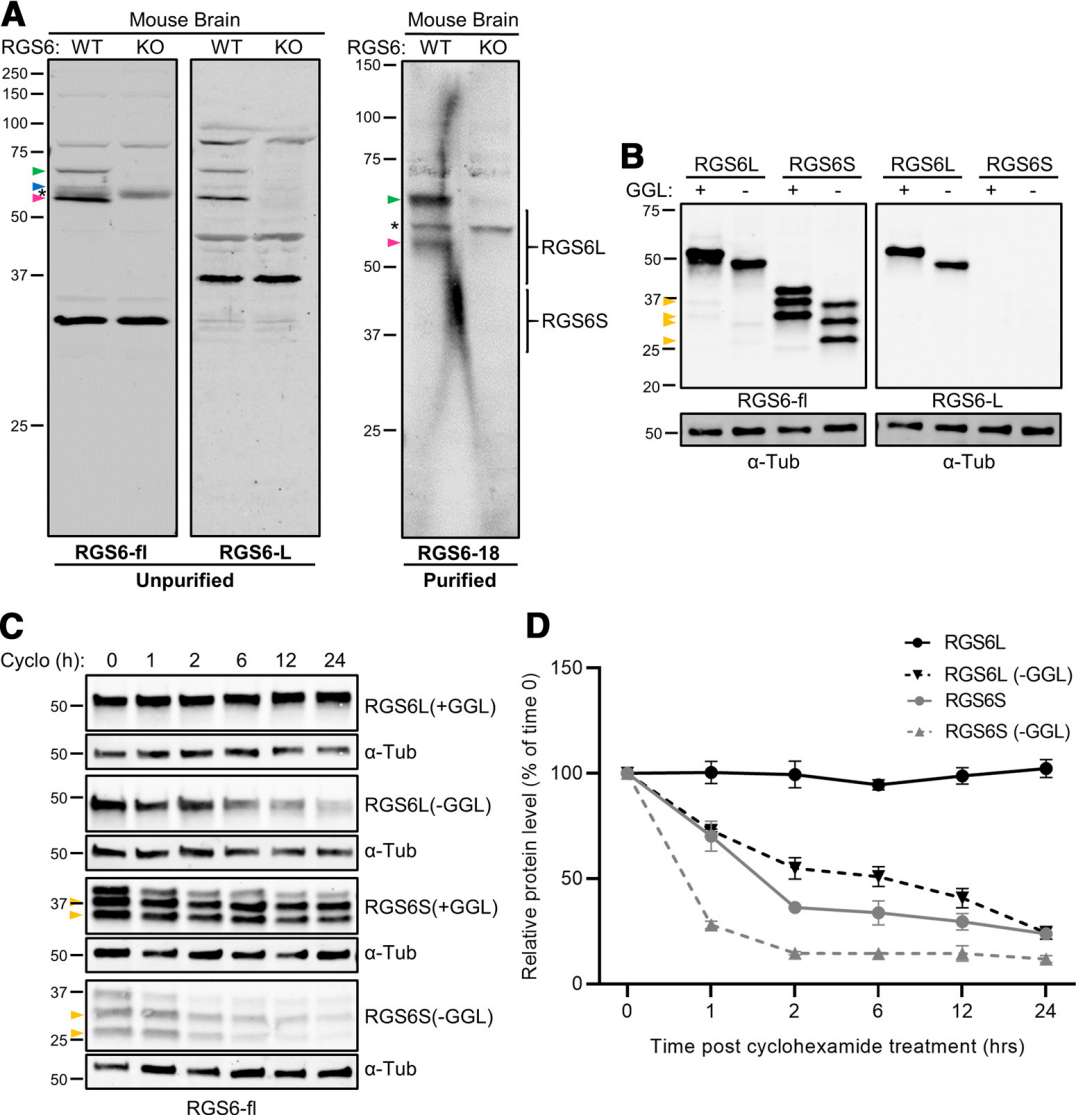
RGS6 antibodies confirm *in vivo* RGS6L(+GGL) expression and identify novel RGS6 protein bands. ***A***, Western blots of whole-brain lysates derived from RGS6^+/+^ (WT) and RGS6^−/−^ (KO) mice using antibodies against: the whole RGS6L protein (RGS6-fl, left panel), the N-terminus of RGS6L protein isoforms (RGS6-L, middle panel), and an 18-amino acid sequence found in 56% of RGS6 protein isoforms (RGS6-18, right panel). Magenta arrows represent RGS6 protein bands corresponding in size to RGS6L(+GGL) isoforms; (Extended Data Fig. 4-1)). Blue and green arrows represent RGS6 protein bands that are larger (61- and 69-kDa) than the predicted sizes (≤57 kDa) of the RGS6 species we previously identified (Chatterjee et al., 2003). Asterisks represents the RGS7 protein band which is non-specifically recognized by the RGS6-fl and RGS6-18 antibodies. ***B***, Western blot analysis demonstrating that while the RGS6-fl antibody recognizes both RGS6L and RGS6S isoforms overexpressed in HEK cells, the RGS6-L antibody specifically recognizes the RGS6L isoforms. Yellow arrows represent additional RGS6 protein bands produced by overexpression of the RGS6L(+/−GGL) and RGS6S(+/−GGL) vector constructs. These bands are smaller than the predicted sizes for the RGS6L(+/−GGL) and RGS6S(+/−GGL) isoforms and likely represent additional protein isoforms produced by novel translation start sites encoded by all constructs (Extended Data Fig. 4-2). ***C***, Representative Western blots from cycloheximide chase experiments characterizing the differential stability of the RGS6L(+/−GGL) and RGS6S(+/−GGL) protein isoforms over a 24 h period. Yellow arrows represent the additional low molecular weight RGS6 protein bands recognized by the RGS6-fl antibody in panel ***B***. These bands were not included in the final analysis. ***D***, Densitometric analysis of protein degradation comparing the four RGS6 protein isoforms (*N *=* *3) in ***C***.

We previously cloned 36 distinct RGS6 mRNA splice forms, from a human brain cDNA library, which were predicted to produce long (RGS6L) and short (RGS6S) RGS6 protein isoforms with an incomplete or intact GGL domain (−/+GGL) and nine alternative C-termini (Fig. 3*A*; Chatterjee et al., 2003). In this study, we endeavored to confirm the existence of these RGS6 isoforms *in vivo*. Therefore, we developed three antibodies, against: the whole RGS6L protein (RGS6-fl), the N-terminus of RGS6L protein isoforms (RGS6-L), and an alternative 18-amino acid sequence found in the C-terminus of 56% of RGS6 isoforms (RGS6-18). Figure 3*B* shows the N-terminal sequence of RGS6 to which the RGS6-L antibody was generated and the lack of sequence conservation in this region with other R7 subfamily members. Figure 3*C* shows the alternatively spliced 18-amino acid sequence to which the RGS6-18 antibody was generated. Because the RGS6-fl antibody was generated to recombinant RGS6Lα2(+GGL) this antibody likely targets multiple epitopes on RGS6 proteins. All antibodies were generated in rabbit against the human protein or its sequences that are conserved in mouse. We examined the ability of these three antibodies to detect native RGS6 protein isoforms by comparing Western blots of whole-brain lysates derived from RGS6^+/+^ [wild-type (WT)] to RGS6^−/−^ [knock-out (KO)] mice (Yang et al., 2010) to identify RGS6-specific bands. Figure 4*A* shows representative Western blots from our studies.

10.1523/ENEURO.0379-21.2021.f4-1 Extended Data Figure 4-1 Predicted molecular weights of known RGS6 isoforms. This table may be used as a reference for determining the putative identities of RGS6 protein bands present on Western blotting. Download Figure 4-1, XLS file.

10.1523/ENEURO.0379-21.2021.f4-2 Extended Data Figure 4-2 Novel putative translation start sites identified within RGS6 protein sequence. Predicted RGS6Lα1 protein sequence. Red star denotes alternative start site utilized to produce RGS6S isoforms. Yellow stars denote novel putative translation start sites present in both RGS6L and RGS6S isoforms. Download Figure 4-2, TIF file.

**Figure 5.**
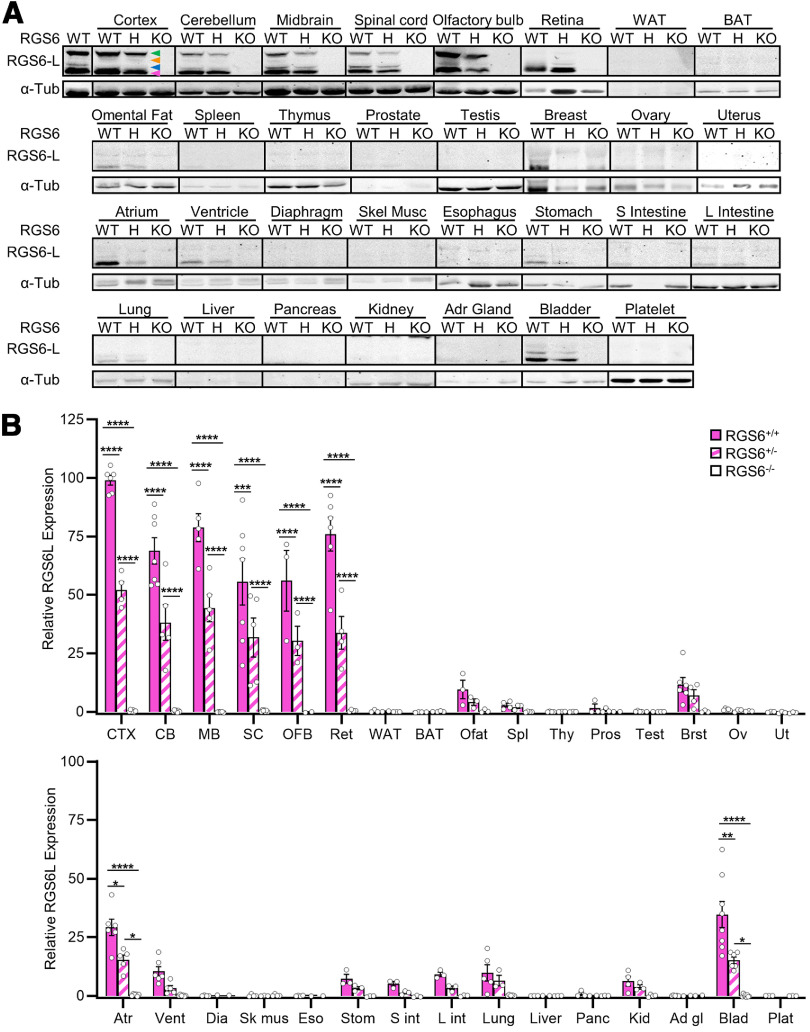
Tissue expression profiling of RGS6 in 31 mouse tissues using the RGS6-L antibody. Using purified RGS6-L antibody we conducted an analysis of RGS6L expression in 31 tissues throughout the mouse body. ***A***, Representative Western blots demonstrating that, in RGS6^+/+^ (WT) and RGS6^+/−^ (H) mice compared with RGS6^−/−^ (KO) mice, many CNS and peripheral tissues express the 53- to 57-kDa RGS6L(+GGL) protein isoforms (magenta arrow) identified previously (Chatterjee et al., 2003). The blue and green arrows indicate the novel 61- and 69-kDa RGS6 protein bands identified in Figure 4*A*, and the orange arrow indicates a third novel 65-kDa band identified in the larger expression profile. While the 65- and 69-kDa RGS6 protein bands were brain-specific, the 61-kDa band was identified in multiple tissues. Note: this figure was constructed from multiple gels. Therefore, we have placed vertical black lines between tissue samples to denote RGS6 expression levels cannot be directly compared between tissues. ***B***, Densitometric quantitative analysis of the 53- to 57-kDa RGS6L(+GGL) isoforms (***A***, magenta arrow) reveal that they are significantly expressed in CNS tissues as well as peripherally in atrium and bladder. Two-way ANOVA found significant effects of strain (*F*_(2,258)_ = 143.75, *p *≤* *0.0001), tissue (*F*_(30,258)_ = 44.11, *p *≤* *0.0001), and their interaction (*F*_(59,258)_ = 15.93, *p *≤* *0.0001). Data were analyzed via two-way ANOVA with Fisher’s LSD *post hoc* adjustment. Data are presented as mean ± SEM; *p ≤* *0.05, **p ≤* *0.01, ***p ≤* *0.001, ****p ≤* *0.0001 (*N *=* *3–8 RGS6^+/+^ mice, 2–6 RGS6^+/−^ mice, and 2–8 RGS6^−/−^ mice). Extended Data Figure 5-1 compares significant differences in RGS6 expression across tissues in RGS6^+/+^ mice. Extended Data Figure 5-2 shows the conservation of an RGS6 SNP in primates. CTX = cerebral cortex, OFB = olfactory bulb, MB = midbrain, CB = cerebellum, SC = spinal cord, Ret = retina, WAT = white adipose tissue, BAT = brown adipose tissue, Ofat = Omental fat, Spl = spleen, Thy = thymus, Pros = prostate, Test = testis, Brst = breast, Ov = ovary, Ut = uterus, Atr = atrium, Vent = ventricle, Dia = diaphragm, Sk mus = skeletal muscle, Eso = esophagus, Stom = stomach, S int = small intestine, L int = large intestine, Panc = pancreas, Kid = kidney, Ad gl = adrenal gland, Blad = bladder, Plat = platelet.

Anti RGS6-fl recognizes three RGS6-specific bands identified by the magenta, blue and green arrows (Fig. 4*A*, left panel). The most abundant of these immunoreactive bands (magenta arrow) has a molecular weight of 53–57 kDa, a size range corresponding to RGS6L protein isoforms containing the GGL domain, required for protein stabilization (RGS6L(+GGL); Extended Data Fig. 4-1; Chen et al., 2003; Cheever et al., 2008; Chatterjee et al., 2003). In addition, RGS6-fl recognizes 61-kDa (blue arrow) and 69-kDa (green arrow) bands that are larger than previously described RGS6 isoforms. We show below that the 69-kDa isoform represents a novel brain-specific form of RGS6L (Fig. 5). Finally, the RGS6-fl antibody also detected a 58-kDa band (Fig. 4*A*, asterisk) in both RGS6^+/+^ and RGS6^−/−^ brain lysates, demonstrating this is not a RGS6 protein isoform. We confirmed by immunoblotting using an RGS7 antibody that this band represents RGS7 (data not shown), the R7 subfamily member most closely related to RGS6. This was unsurprising given that RGS6-fl was generated to recombinant RGS6L.

10.1523/ENEURO.0379-21.2021.f5-1 Extended Data Figure 5-1 Comparison of RGS6 expression across 31 mouse tissues using the RGS6-L antibody. Using the RGS6+/+ (WT) tissue data from Figure 5, we have graphed RGS6 protein expression detected with the RGS6-L antibody across the same 31 tissues. Here, we find that RGS6 is most highly expressed in the cerebral cortex (CTX, p ≤ 0.000) relative to all other tissues tested. Data are represented as mean ± SEM. Data were analyzed via one-way ANOVA with Fisher’s LSD post hoc adjustment. Table below displays the p values of all comparisons of RGS6 expression across the various tissues. CTX = cerebral cortex, OFB = olfactory bulb, MB = midbrain, CB = cerebellum, SC = spinal cord, Ret = retina, WAT = white adipose tissue, BAT = brown adipose tissue, Ofat = Omental fat, Spl = spleen, Thy = thymus, Pros = prostate, Test = testis, Brst = breast, Ov = ovary, Ut = uterus, Atr = atrium, Vent = ventricle, Dia = diaphragm, Sk mus = skeletal muscle, Eso = esophagus, Stom = stomach, S int = small intestine, L int = large intestine, Panc = pancreas, Kid = kidney, Ad gl = adrenal gland, Blad = bladder, Plat = platelet. Download Figure 5-1, TIF file.

10.1523/ENEURO.0379-21.2021.f5-2 Extended Data Figure 5-2 Evolutionary conservation of the rs2332700 SNP. Human RGS6 sequence (37 bps) flanking the rs2332700 SNP (G > C) aligned against the corresponding sequence present in 30 different animal species across the animal kingdom using the UCSC Genome Browser. The SNP is only found in primates. =: gap regions in genomes, in which aligning species has one or more unalignable bases. This is likely due to excessive evolutionary distance between the aligning species and human or due to independent insertions/deletions in the region between the aligning species and human. Download Figure 5-2, TIF file.

Interestingly, while protein bands corresponding to RGS6L(+GGL) were readily apparent when using the RGS6-fl antibody isoforms lacking the GGL domain (RGS6L(–GGL), 49–52 kDa; Extended Data Fig. 4-1) were not (Fig. 4*A*, left panel) even with long blot exposures (data not shown). Because the RGS6-fl antibody can detect RGS6L(–GGL) isoforms expressed in HEK 293 cells (Fig. 4*B*), the apparent absence of these isoforms *in vivo* is likely because of their relative instability compared with RGS6L(+GGL) isoforms, as suggested by cycloheximide chase experiments in HEK cells (Fig. 4*C*,*D*), and/or low abundance when compared with RGS6L(+GGL) isoforms. Interestingly, the RGS6-fl antibody also did not detect RGS6S(+/−GGL) proteins (34–41 kDa; Extended Data Fig. 4-1) in mouse brain (Fig. 4*A*, left panel), although RGS6S transcripts were originally cloned from a human brain cDNA library (Chatterjee et al., 2003). As with the RGS6L(–GGL) isoforms, the inability to detect the RGS6S isoforms is not because of a lack of antibody specificity because anti-RGS6-fl readily detects RGS6S(+/−GGL) proteins following expression in HEK293 cells (Fig. 4*B*). Therefore, lack of RGS6S isoforms *in vivo* may be due once again to their relative instability, suggested by cycloheximide chase experiments (Fig. 4*C*,*D*), and/or low their abundance when compared with RGS6L isoforms.

As the RGS6-fl antibody recognized not only RGS6, but also RGS7, a second antibody, RGS6-L, was generated against the first 19 amino acids (MAQGSGDQRAVGVADPEES) of the RGS6L isoforms, a sequence that shows minimal conservation across the R7 subfamily (Fig. 3*B*). The caveat to the RGS6-L antibody is that it does not detect RGS6S(+/−GGL) isoforms (Fig. 4*B*) which have a truncated N-terminus (Fig. 3*A*). However, given that the RGS6-fl antibody did not detect endogenous RGS6S isoforms in mouse brain (Fig. 4*A*, left panel), or in any other mouse peripheral tissues tested (data not shown), use of the RGS6-L antibody was deemed an acceptable alternative strategy. Subsequent Western blot validation of the RGS6-L antibody revealed that it detects the same three RGS6 protein bands (magenta, green, and blue arrows) as theRGS6-fl antibody, but, unlike the RGS6-fl antibody, it does not detect RGS7 (Fig. 4*A*, middle panel, asterisk). Again, the most abundant immunoreactive band detected with both RGS6L and RGS6-fl antibodies is the 53- to 57-kDa band (magenta arrow), corresponding to RGS6L(+GGL) isoforms (Extended Data Fig. 4-1), followed by the brain-specific 69-kDa band (green arrow) and then the 61-kDa band (Fig. 4*A*, left and middle panels, blue arrow). No other RGS6 proteins are detected by these two antibodies.

While the RGS6-L antibody eliminates detection of RGS7, it does not allow for easy delineation between the nine RGS6L(+GGL) isoforms, as they fall within a narrow molecular weight range (53–57 kDa; Extended Data Fig. 4-1). Therefore, a third antibody, RGS6-18, was generated against the alternative PESEQGRRTSLEKFTRSV amino acid sequence (Fig. 3*C*) encoded by exon 18 present near the C-terminus of 56% of RGS6 isoforms (Fig. 3*A*, dark gray box starting at amino acid 456). As the RGS6-L and RGS6-18 antibodies were both made in rabbit, it was not possible to directly compare their banding pattern in a single blot. However, Western blot analysis using RGS6-18 shows strong immunoreactivity of the 69-kDa brain-specific band (green arrow) and the 53- to 57-kDa band (magenta arrow) corresponding to RGS6L(+GGL) isoforms (Fig. 4*A*, right panel). The RGS6-18 antibody also recognizes RGS7 (Fig. 4*A*, right panel, asterisk) as a portion of the alternative amino acid sequence (RRTSLEKFTRSV) is present (≤75% conservation) in a subset of RGS7 isoforms.

In summary, Western blot analysis of RGS6 isoform expression in mouse brain tissue reveals that two of the antibodies, RGS6-fl and RGS6-18, also detect RGS6’s closest evolutionary relative, RGS7. However, the RGS6-L antibody generated against the N-terminal sequence unique to RGS6L isoforms eliminates this problem. Further analysis revealed that while RGS6 proteins with molecular weights corresponding to RGS6L(+GGL) isoforms were readily detectable, RGS6L(−GGL) and RGS6S(+/−GGL) isoforms were not, possibly because of their relative instability or low abundance.

There are two remaining points of interest that arose from these initial cell culture and mouse tissue experiments. First, we discovered that overexpression of RGS6L(+/−GGL) and RGS6S(+/−GGL) transcripts in culture results in the expression of several protein bands, detected by the RGS6-fl antibody, that are not only equivalent in size but also smaller than the predicted protein molecular weights (Fig. 4*B*, yellow arrows, *C*). Subsequent analysis of both transcript sequence and protein band molecular weights suggest that the small bands are likely RGS6 protein isoforms produced through alternative start sites. These alternative start sites are present in all four overexpressed transcripts, and thus similar low molecular weight bands are produced by each. As these alternative start sites exist in exons 8 and 10 (Extended Data Fig. 4-2, yellow stars), present in all RGS6L and RGS6S transcripts, we would predict that these low molecular weight RGS6 proteins may be expressed in any tissue expressing RGS6. Second, Western blots of mouse whole-brain lysate revealed at least two additional RGS6 protein bands that were larger (61- and 69-kDa, blue and green arrows, respectively) than all previously described isoforms (Fig. 4*A*, left and middle panels). Both protein bands were detected by the RGS6-fl and RGS6-L antibodies, but only the 69-kDa protein band was detected by the RGS6-18 antibody (Fig. 4*A*, right panel). From these data, the significance of these bands was uncertain. They are bona fide RGS6 protein isoforms as they were not detected in RGS6^−/−^ mice, but whether they represent novel RGS6 isoforms or posttranslational modified proteins remained unclear.

### RGS6L(+GGL) is most highly expressed in CNS but is also expressed in several peripheral tissues

As the RGS6-L antibody does not recognize RGS7, we used it to conduct a comprehensive Western blot analysis of RGS6L expression in 31 tissues throughout the mouse body (Fig. 5). To standardize the Westerns and allow for quantitative analysis of RGS6 expression across tissues, one RGS6^+/+^ whole-brain lysate was included as a control/constant denominator in every blot. These blots, and the subsequent densitometric analysis, revealed that RGS6L is significantly expressed in all CNS tissues analyzed, including, but not limited to the: cerebral cortex, olfactory bulb, midbrain, cerebellum, spinal cord, and retina (Fig. 5). Furthermore, RGS6L isoforms were also found at significant, albeit lower levels (Fig. 5; Extended Data Fig. 5-1) in two peripheral mouse tissues (heart and bladder). Finally, while statistical significance was not achieved because of small sample size, tissue variability, or low level of expression, RGS6 protein bands were also identified in lung, kidney, prostate, heart, omental fat, stomach, intestines, and breast (Fig. 5). This RGS6 expression profile corresponds well with previous reports describing the physiological and pathophysiological roles of RGS6 (Fig. 1).

**Figure 6.**
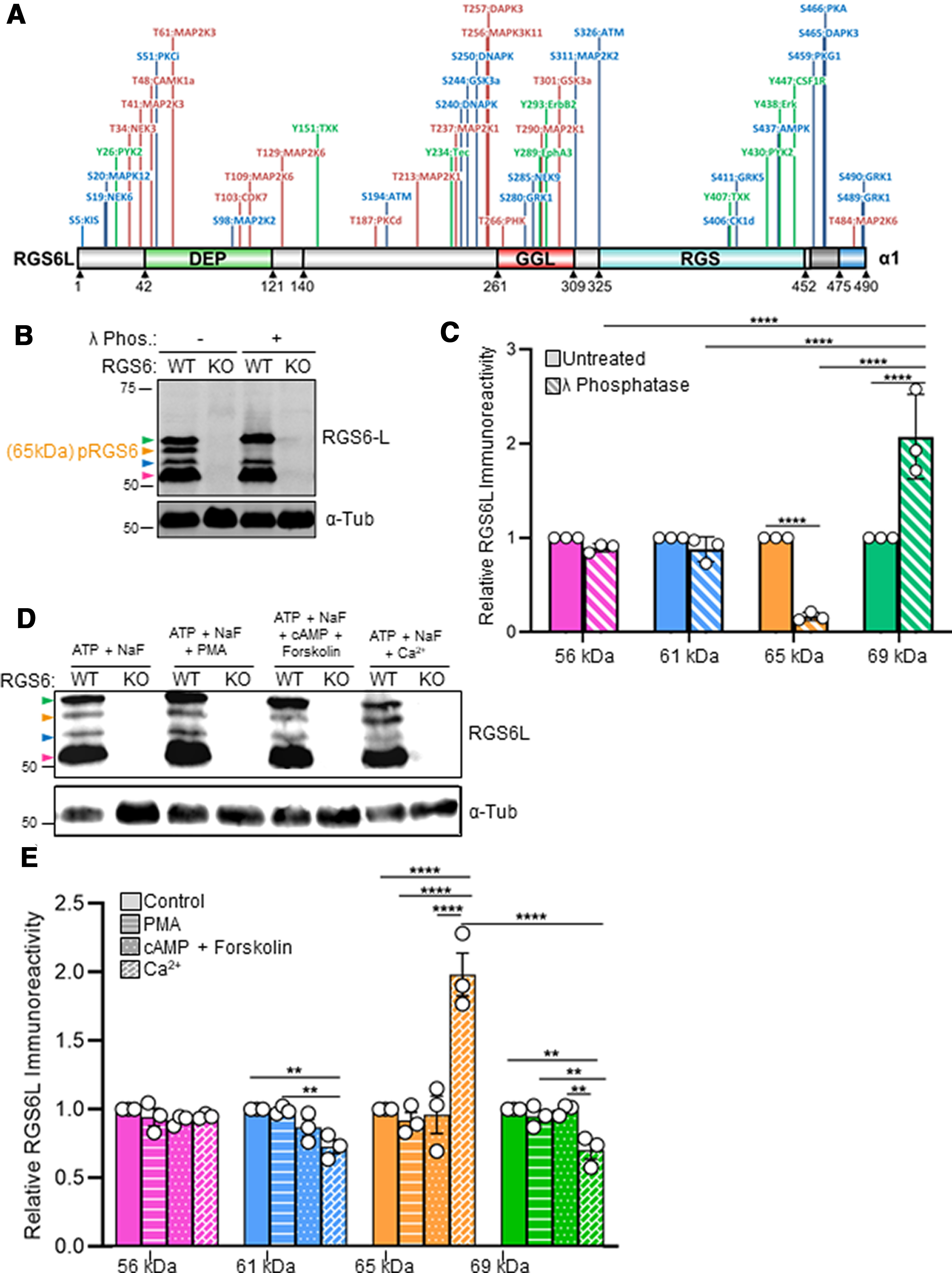
Immunohistochemical analysis of RGS6 tissue distribution. Representative images from an immunohistochemical analysis of RGS6 protein distribution, using the RGS6-fl antibody, in mouse RGS6^+/+^ (WT) CNS (left panel) and peripheral (right panel) tissues shown to express RGS6 protein bands via Western (Fig. 5*A*). Complimentary tissues collected from RGS6^−/−^ (KO) controls confirmed that the RGS6-fl antibody, while it recognizes RGS7 via Western blots (Fig. 4*A*), is specific for RGS6 in tissue sections.

To compliment this broad expression profile, we conducted an immunohistochemical analysis of RGS6 distribution (Fig. 6) in tissues found to express RGS6 protein bands via Western blot (Fig. 5). The RGS6-fl antibody was used for this analysis, instead of the RGS6-L antibody, as it proved to stain tissues more reliably (data not shown). Furthermore, simultaneous staining of both RGS6^−/−^ and RGS6^+/+^ tissues demonstrated that, unlike with Western blotting, the RGS6-fl antibody does not detect RGS7 proteins in intact tissues (Fig. 6). In the CNS, this analysis revealed that RGS6 is expressed ubiquitously across the cortical layers and confirmed what we had previously reported in the cerebellum (Maity et al., 2012), that RGS6 is expressed predominantly by neurons in the granule cell layer and Purkinje neurons. Likewise, there is a predominant neuronal expression of RGS6 in the spinal cord and olfactory bulb, as well as the midbrain, the latter finding confirming our previous evidence that RGS6 is expressed in midbrain dopaminergic neurons (Bifsha et al., 2014; Luo et al., 2019). Finally, in the retina, RGS6 proteins are highly expressed in ganglion cells as well as retinal pigmented epithelium and to a lesser extent in bipolar cells, rods, and cones. Moving to the periphery, robust RGS6 expression is detected throughout the walls of the heart, in omental fat cell plasma, and in epithelial cells of lung, bladder, breast, intestines, stomach, and kidney. A lower RGS6 expression is also seen in connective/muscular tissues adjacent to epithelial cells (Fig. 6).

Finally, an in-depth analysis of the RGS6 expression profile blots also elucidated the most prevalent RGS6L isoforms. Consistent with what had been seen earlier in whole-brain lysates, the molecular weight of the protein bands observed suggested that RGS6L(+GGL) isoforms predominate (magenta arrow, 53 to 57-kDa) in all tissues examined. Furthermore, it became apparent that, in addition to the large 61- and 69-kDa bands (blue and green arrows, respectively), the RGS6-L antibody also detected a third novel protein band of ∼65-kDa (orange arrow) in RGS6^+/+^ and RGS6^+/−^, but not RGS6^−/−^, mice. This band, while faint, was clearly visible in most CNS tissues blots with longer exposure times. Examination of the expression profile for all three bands revealed that while the 61-kDa band is expressed in multiple tissues (brain, omental fat, breast, heart, stomach, intestine, lung, and bladder) the 65- and 69-kDa bands are brain-specific (Fig. 5). Once again, the significance of these findings, whether these bands represented novel isoforms or posttranslationally modified proteins, remained unclear. However, considering RGS6’s association with various neuropsychiatric disorders, we were very intrigued by the discovery that two of these novel RGS6 protein bands were brain-specific.

### The novel, 65-kDa brain-specific RGS6 is a phosphorylated protein

**Figure 7.**
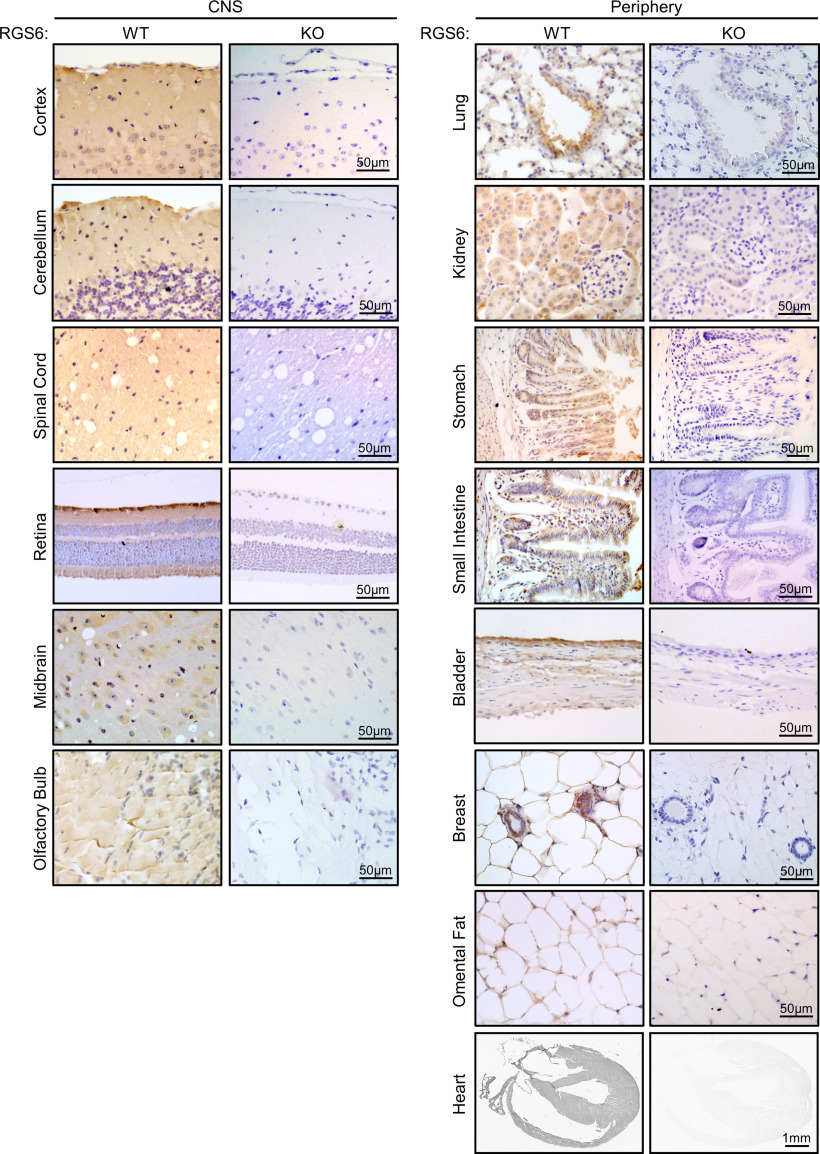
Prediction of RGS6Lα1(+GGL) phosphorylation sites and identification of a novel, ∼65-kDa brain-specific RGS6 phosphoprotein. ***A***, Putative phosphorylation sites within the RGS6Lα1(+GGL) protein were identified using the GPS 3.0 software (Extended Data Fig. 7-1). Sites with the highest prediction score were mapped to the RGS6Lα1(+GGL) protein sequence with the corresponding kinase labeled. Serine residues are indicated in blue, threonine in pink, and tyrosine in green. ***B***, A representative Western blot from RGS6^+/+^ (WT) and RGS6^−/−^ (KO) brain tissue lysates treated where indicated with λ phosphatase. The magenta arrow indicates the ∼53- to 57-kDa RGS6L(+GGL) isoforms, while the blue, orange, and green arrows indicate the novel RGS6 protein bands (61-, 65-, 69-kDa, respectively) identified in this study. ***C***, A densitometric quantification of the 56-, 61-, 65-, and 69-kDa RGS6 protein bands (Column colors correspond to arrow colors in Western, ***B***). Two-way ANOVA with Fisher’s LSD *post hoc* adjustment revealed a significant effect of λ phosphatase on the expression of the 65-kDa (*p *≤* *0.0001) and 69-kDa (*p *≤* *0.0001) bands relative to their respective untreated samples. Data are presented as mean ± SEM; ****p ≤* *0.0001 (*N *=* *3 mice per treatment). ***D***, A representative Western blot from RGS6^+/+^ (WT) and RGS6^−/−^ (KO) brain tissue lysates treated where indicated with PMA (200 nm), cAMP and Forskolin (10 μm of each), or Ca^2+^(10 μm). ***E***, A densitometric quantification of the 56-, 61-, 65-, and 69-kDa RGS6 protein bands (Column colors correspond to arrow colors in Western, ***D***). Two-way ANOVA with Fisher’s LSD *post hoc* adjustment found a significant effect of Ca^2+^ treatment on the expression of the 65-kDa (*p *≤* *0.0001) and 69-kDa (*p *=* *0.002) bands relative to their respective untreated samples.

Phosphorylation is a common form of posttranslational modification. Indeed, the R7 family members, RGS7 and RGS9, have previously been shown to be phosphorylated (Benzing et al., 1999, 2002; Balasubramanian et al., 2001; Sokal et al., 2003; Garzón et al., 2005; Ibi et al., 2011). There is also evidence from large-scale proteomic screens of the murine brain that supports the existence of RGS6 phospho-peptides with modifications on S244, S437, S459, and S490 of RGS6Lα1 (Huttlin et al., 2010; Safaei et al., 2011; Trinidad et al., 2012). Furthermore, group-based prediction of protein phosphorylation using GPS 3.0 software indicates that there are potentially 46 threonine, serine, and tyrosine residues in the RGS6Lα1 (+GGL) protein that can serve as kinase substrates (Fig. 7*A*; Extended Data Fig. 7-1). Several of these residues were also identified as sites of potential phosphorylation via a second, similar program, PhosphoNET, as well (Extended Data Fig. 7-1). Therefore, we hypothesized that the large novel RGS6L protein bands may represent phospho-proteins and subsequently performed an *in vitro* phosphatase assay using protein lysates prepared from the whole RGS6^+/+^ and RGS6^−/−^ mouse brains. This assay revealed that addition of λ phosphatase to the lysate mixture eliminated the 65-kDa RGS6L protein band, but not the 61- and 69-kDa RGS6L species (Fig. 7*B*). This assay suggests that the 65-kDa RGS6L protein band (orange arrow) represents a brain-specific phospho-protein.

10.1523/ENEURO.0379-21.2021.f7-1 Extended Data Figure 7-1 Putative phosphorylation sites in the RGS6Lα1 protein. X denotes sites that were also predicted by PhosphoNet or have been confirmed as phosphorylated residues through mass spectrometry datasets. Download Figure 7-1, XLS file.

Additional experiments revealed that the 65- and 69-kDa RGS6 isoforms represent interconvertible phospho and dephospho forms of RGS6, respectively. First, in support of this, analysis of the effects of phosphatase treatment on RGS6 isoforms revealed that phosphatase-induced loss of the 65-kDa band was accompanied by an increase in the 69-kDa band (Fig. 7*C*). This suggested that dephosphorylation of the 65-kDa band did not result in its disappearance, but instead promoted an upward shift in its electrophoretic mobility to 69 kDa. Although phosphorylation does not change the electrophoretic mobility of most proteins, it can lead to an upward or downward shift in mobility depending on the protein. Our results suggested that dephosphorylation of the 65-kDa form produced an upward mobility shift. Noteworthy examples of proteins showing upward mobility shifts on phosphorylation include cyclin-dependent kinase-2 (Cdk-2; Gu et al., 1992), Cdk1 (Larochelle et al., 1998), and Cdk-7 (Garrett et al., 2001). To confirm this, we performed kinase reactions in brain extracts with ATP and various kinase activators. Figure 7*D*,*E* show that incubation of extracts with ATP and Ca^2+^ promoted an increase in the 65-kDa RGS6 band and a decrease in the 69-kDa RGS6 band. These findings corroborate our evidence from Figure 7*B*,*C* that the 65-kDa band is a phosphorylated form of RGS6 and that dephosphorylation leads to an upward mobility shift to the 69-kDa band.

The predicted size of the largest RGS6 isoform shown in Figure 7*A* is 56.5-kDa (Extended Data Fig. 4-1). Our studies shown in Figure 7*B–E* show that the 53- to 57-kDa and the 61-kDa isoforms are not affected by phosphatase treatment or by incubation with ATP and kinase activators. Yet, all known members of the RGS6 protein family have some or all of the sequence elements shown in Figure 7*A*. It is possible that the expected additional protein sequence in the novel brain-specific 69-kDa possesses the site for Ca^2+^-dependent phosphorylation. Alternatively, it may possess sequence elements that confer substrate availability of sequences present in all/most RGS proteins shown in Figure 7*A*. It is also possible that the 53- to 57-kDa isoforms do not undergo mobility shifts on phosphorylation/dephosphorylation and that the 61-kDa isoform represents a novel RGS6 isoform whose transcript was not identified in the initial cloning effort (Chatterjee et al., 2003).

### The novel, 69-kDa brain-specific RGS6 isoform is highly conserved and significantly expressed in all regions of the brain analyzed

**Figure 8.**
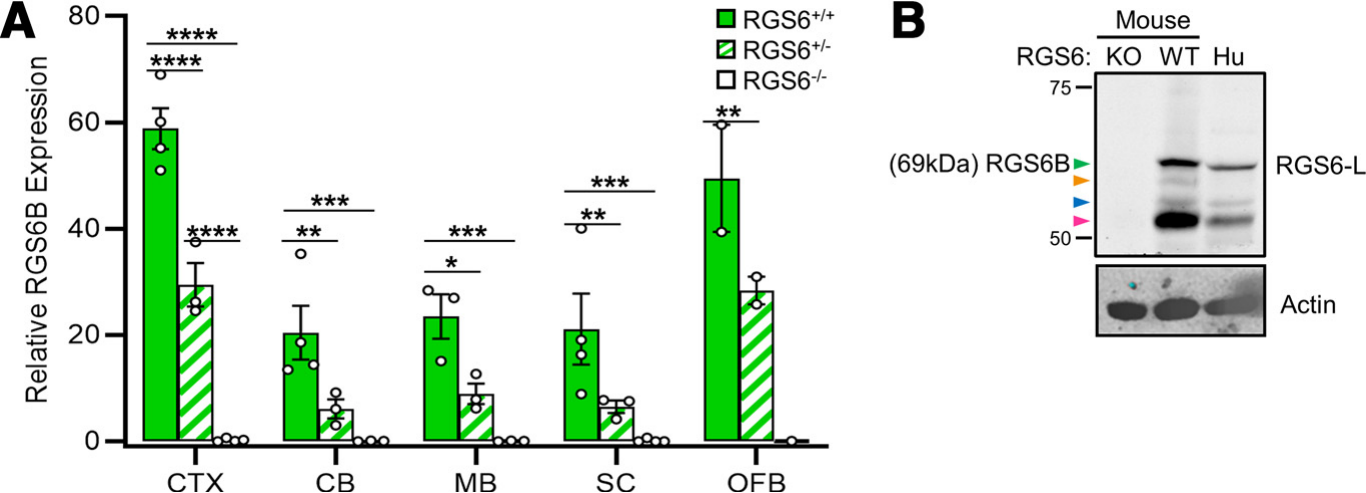
The 69-kDa brain-specific RGS6 protein band (RGS6B) is highly conserved and significantly expressed in all mouse CNS tissues analyzed. ***A***, Densitometric quantification of the 69-kDa, brain-specific RGS6 protein band (Fig. 5*A*, green arrow) demonstrates that it is significantly expressed in all RGS6^+/+^ (WT) mouse brain tissues, compared with RGS6^+/−^ (H) and RGS6^−/−^ (KO) tissues analyzed. Two-way ANOVA found significant effects of strain (*F*_(2,32)_ = 99.51, *p *≤* *0.0001), tissue (*F*_(4,32)_ = 21.19, *p *≤* *0.0001), and their interaction (*F*_(8,32)_ = 21.19, *p *=* *0.000). Data were analyzed via two-way ANOVA with Fisher’s LSD *post hoc* adjustment. Data are presented as mean ± SEM; *p ≤* *0.05, **p ≤* *0.01, ***p ≤* *0.001, ****p ≤* *0.0001 (*N *=* *3–4 RGS6^+/+^ mice, 2–3 RGS6^+/−^ mice, and 3–4 RGS6^−/−^ mice). Extended Data Figure 8-1 compares significant differences in RGS6B expression across tissues in RGS6^+/+^ mice. CTX = cerebral cortex, CB = cerebellum, MB = midbrain, SC = spinal cord, OFB = olfactory bulb. ***B***, Western blot analysis of mouse and human brain lysates indicate that the 61- and 69-kDa, brain-specific protein bands (blue and green arrows, respectively) are conserved in mouse and human.

We found that the 69-kDa dephospho form of RGS6 was generally easier to detect and quantify in various brain regions than the 65-kDa form, possibly because of dephosphorylation of the 65-kDa isoform following sacrifice and brain dissection. The function of these isoforms in brain is unclear, but a cursory analysis hints at their importance. First, quantification of the 69-kDa band demonstrates that it is significantly expressed, in all RGS6^+/+^ mouse brain tissues assayed (Fig. 8*A*; Extended Data Fig. 8-1), although its expression level appears to be consistently lower than that of the 53- to 57-kDa RGS6L protein isoforms (Fig. 7*B*,*D*). Given its exclusive expression in brain tissue we hereafter refer to the 69-kDa isoform as RGS6B (and the 65-kDa form as p-RGS6B). Second, RGS6B appears to serve an important evolutionary function as Western blot analysis reveals that it is not only expressed in mouse brain tissue but also human brain tissue (Fig. 8*B*, green arrow).

10.1523/ENEURO.0379-21.2021.f8-1 Extended Data Figure 8-1 Comparison of RGS6B expression across CNS mouse tissues using the RGS6-L antibody. Using the RGS6+/+ (WT) tissue data from Figure 8, we have graphed RGS6B protein expression detected with the RGS6-L antibody across mouse CNS tissues. Data are represented as mean ± SEM. Data were analyzed via one-way ANOVA with Fisher’s LSD post hoc adjustment. Table below displays the p values of all comparisons of RGS6 expression across the various tissues. CTX = cerebral cortex, CB = cerebellum, MB = midbrain, SC = spinal cord, OFB = olfactory bulb. Download Figure 8-1, TIF file.

### RGS6 is the only R7 family member expressed in lung, omental fat, bladder, stomach, and intestine

**Figure 9.**
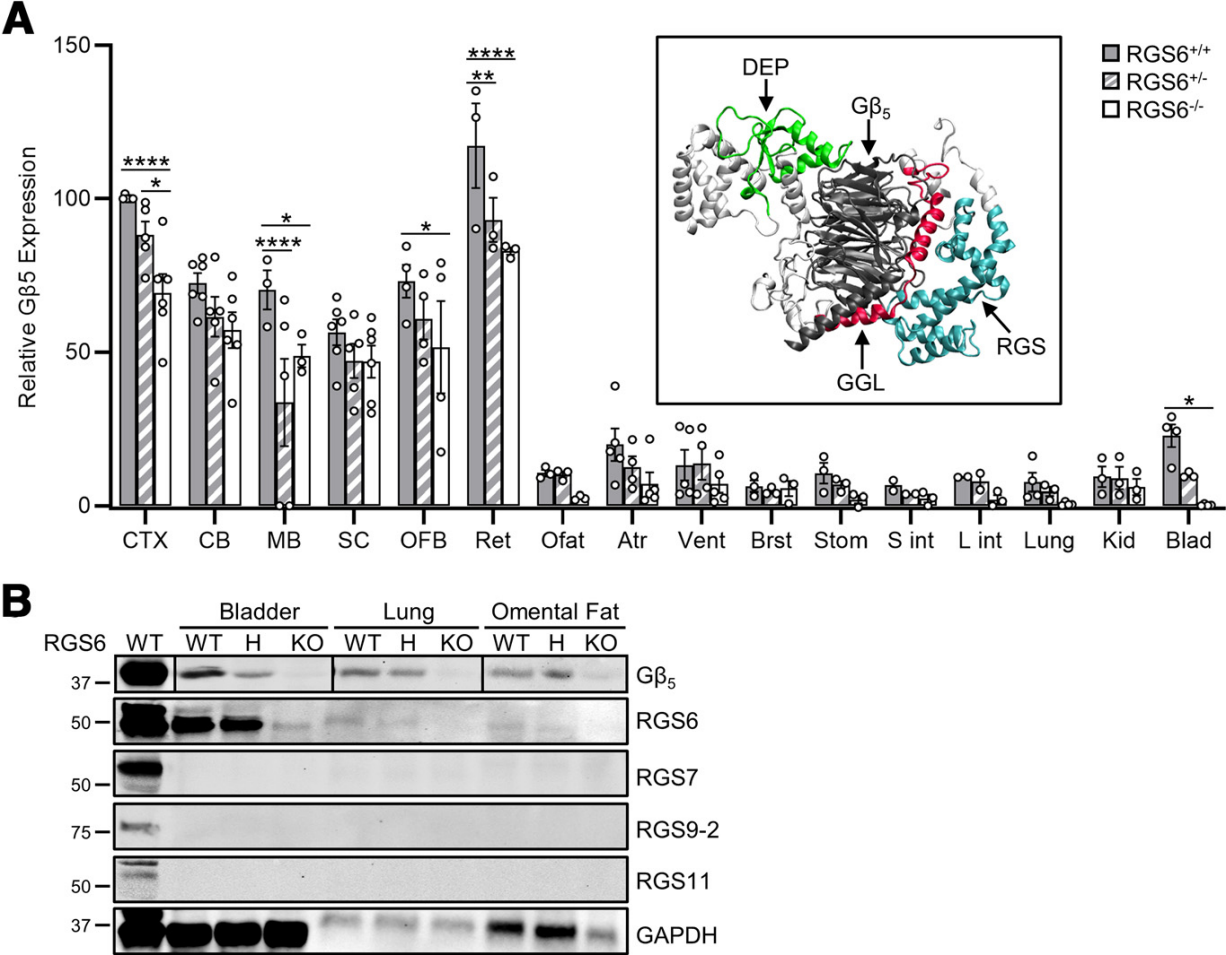
RGS6 is the only R7 family member expressed in bladder, lung, omental fat, stomach, and intestine. ***A***, Densitometric quantification of Gβ_5_ expression in tissues shown to express RGS6 protein bands via Western (Fig. 5*A*). Inset, Structure homology modeling of RGS6Lα2(+GGL) in complex with Gβ_5_. Two-way ANOVA found significant effects of strain (*F*_(2,135)_ = 19.69, *p *≤* *0.0001) and tissue (*F*_(15,135)_ = 90.64, *p *≤* *0.0001). Data were analyzed via two-way ANOVA with Fisher’s LSD *post hoc* adjustment. Data are presented as mean ± SEM; *p ≤* *0.05, **p ≤* *0.01, ***p ≤* *0.001, ****p ≤* *0.0001 (*N *=* *2–6 RGS6^+/+^ mice, 2–5 RGS6^+/−^ mice, and 3–6 RGS6^−/−^ mice). Extended Data Figure 9-1 compares significant differences in Gβ_5_ expression across tissues in RGS6^+/+^ mice. CTX = cerebral cortex, CB = cerebellum, MB = midbrain, SC = spinal cord, OFB = olfactory bulb, Ret = retina, Ofat = Omental fat, Atr = atrium, Vent = ventricle, Brst = breast, Stom = stomach, S int = small intestine, L int = large intestine, Kid = kidney, Blad = bladder. ***B***, Representative Western blot analysis of a subset of the tissues from ***A*** showing a concomitant loss of RGS6 and Gβ_5_ expression using antibodies against the individual R7 family members confirmed that RGS6 is the only R7 member expressed there. Note: The Gβ_5_ portion of this figure was constructed from multiple blots. Therefore, we have placed vertical black lines between Gβ_5_ tissue samples to denote expression levels cannot be directly compared. The first lane labeled WT is a lysate from whole brain for all blots except for the blot for RGS9-2, where it is striatum, as RGS9-2 is not detectable in whole-brain lysates (Luo et al., 2019). WT = RGS6^+/+^, H = RGS6^+/−^, and KO = RGS6^−/−^.

As mentioned earlier, the Western blot profile of RGS6L protein expression (Fig. 5) was consistent with the literature regarding RGS6’s physiological and pathophysiological roles (Fig. 1). However, previous analysis of RGS mRNA expression profiles indicate that there is often overlap in the expression of several RGS’s within a single tissue. For example, *in situ* hybridization (Gold et al., 1997) and RT-qPCR analyses (Larminie et al., 2004) demonstrated that the entire R7 RGS subfamily is expressed in the brain. Therefore, to determine if, in tissues where it is expressed, RGS6 is the primary R7 subfamily member present we re-probed the RGS6-L Western blots (Fig. 5) with an antibody against Gβ_5_ (Fig. 9). Gβ_5_ expression was found to be significantly higher in brain and retina versus other tissues (Extended Data Fig. 9-1) as observed for RGS6L (Extended Data Fig. 5-1).

10.1523/ENEURO.0379-21.2021.f9-1 Extended Data Figure 9-1 Comparison of Gβ5 expression across 16 mouse tissues. Using the RGS6+/+ (WT) tissue data from Figure 9, we have graphed Gβ5 protein expression across 16 mouse tissues. Data are represented as mean ± SEM. Data were analyzed via one-way ANOVA with Fisher’s LSD post hoc adjustment. Table below displays the p values of all comparisons of RGS6 expression across the various tissues. CTX = cerebral cortex, CB = cerebellum, MB = midbrain, SC = spinal cord, OFB = olfactory bulb, Ret = retina, Ofat = Omental fat, Atr = atrium, Vent = ventricle, Brst = breast, Stom = stomach, S int = small intestine, L int = large intestine, Kid = kidney, Blad = bladder. Download Figure 9-1, TIF file.

Gβ_5_ is an atypical Gβ subunit that forms an obligate dimer with all R7 family members through interaction with their GGL domain (Fig. 3), which is structurally homologous to conventional Gγ subunits. Interaction between Gβ_5_ and R7 family members is obligatory, loss of either interacting partner causes destabilization of the other (Chen et al., 2003). Emphasizing this point, in 2008, Cheever and colleagues solved the crystal structure of RGS9 and showed it in complex with Gβ_5_ (Cheever et al., 2008). Furthermore, this crystal structure revealed that the interaction between RGS9’s GGL domain and Gβ_5_ mirrors the orientation and interaction of the conventional Gβγ dimer. This interaction orientation is believed to be maintained throughout the R7 subfamily, a hypothesis which is supported both by the solving of the crystal structure for RGS7, also in complex with Gβ_5_ (Patil et al., 2018), but also through structure homology modeling of RGS6Lα2(+GGL) (Fig. 9*A*, inset). Therefore, loss of Gβ_5_ expression with RGS6 removal would indicate that RGS6 is the only R7 family member present in that tissue.

These analyses demonstrated that, while most tissues expressing RGS6 also express other R7 family members (Gβ_5_ expression was maintained after RGS6 loss), there were some tissues, including lung, bladder, stomach, intestine, and omental fat, where RGS6 appeared to be the only R7 family member present (Gβ_5_ expression was completely lost with loss of RGS6; Fig. 9*A*). Indeed, Western blot analysis of a subset of the tissues showing a concomitant loss of RGS6 and Gβ_5_ expression (lung, omental fat, and bladder) using antibodies against individual R7 family members (RGSs 7, 9–2, and 11) confirmed that RGS6 is the only R7 member expressed there (Fig. 9*B*).

## Discussion

RGS6 pre-mRNA is subject to alternative splicing producing 36 unique transcripts each predicted to produce viable protein isoforms (Chatterjee et al., 2003). Despite knowing about this complex processing, sequence similarities have complicated the study of individual RGS6 isoforms. As such, most research implicating RGS6 in neuropsychiatric disorders or exploring its diverse peripheral roles have taken a global view of its function. This work provides the first comprehensive analysis of RGS6 isoform expression. Using three distinct antibodies generated against the whole RGS6L protein, the N-terminus of RGS6L isoforms, and an alternative amino acid sequence found in the C-terminus of 56% of RGS6 isoforms, we have not only begun to define tissue-specific differences in RGS6 isoform expression but have also identified brain-specific RGS6 protein bands that appear to be phosphorylated/dephosphorylated only in CNS tissues.

### RGS6L(+GGL) isoforms are the most prevalent isoforms expressed *in vivo*

Western blot analyses demonstrate that RGS6L(+GGL) isoforms predominate throughout the mouse body (Fig. 5, magenta arrow). As the nine possible RGS6L(+GGL) isoforms fall within a narrow molecular weight range (53–57 kDa; Extended Data Fig. 4-1), we were unable to definitively say which of these isoforms were present. However, using the RGS6-18 antibody, we conclude that, in the brain, at least some of the RGS6L(+GGL) isoforms include the amino acid sequence encoded by exon 18 (Fig. 4*A*, right panel). Furthermore, we hypothesize that RGS6Lα1(+GGL) and RGS6Lα2(+GGL), which differ only in the inclusion and exclusion, respectively, of the amino acid sequence encoded by exon 18 (Fig. 3), may represent the predominant brain RGS6L(+GGL) isoforms. This hypothesis is supported by the fact that RGS6Lα2(+GGL) (GenBank AF073920) and RGS6Lα1(+GGL) (GenBank AF107619) were the first RGS6L splice forms identified. These results suggest that RGS6Lα1(+GGL) and RGS6Lα2(+GGL) are expressed at relatively high levels compared with other RGS6L(+GGL) transcripts which were not identified until our subsequent cloning study (Snow et al., 1999; Chatterjee et al., 2003).

While the studies above suggest certain RGS6L(+GGL) isoforms may predominate because of relatively high mRNA transcript expression, this is likely not the only explanation for their prevalence. The R7 RGS protein subfamily is distinguished by two unique domains outside of the RGS domain: the DEP/DHEX domain and the GGL domain. Importantly, the GGL domain is required for interaction with Gβ_5_, an atypical Gβ subunit that forms an obligate dimer with all R7 family members, promoting their stability (Chen et al., 2003). Consequently, RGS6L(+GGL) isoforms may be expected to be more stable (Fig. 4*D*) and thus expressed at higher levels relative to the RGS6L(–GGL) isoforms, which we previously showed do not bind Gβ5 (Liu and Fisher, 2004).

Interestingly, as with the RGS6L(–GGL) isoforms, we did not identify RGS6 protein bands in the brain corresponding in size to RGS6S isoforms (Fig. 4*A*; Extended Data Fig. 4-1), despite the fact their corresponding transcripts were originally cloned from a human brain cDNA library (Chatterjee et al., 2003). While this may be explained by low transcript abundance, it may also be caused by protein instability as suggested by our cycloheximide chase experiments (Fig. 4*C*,*D*). RGS6L and RGS6S protein isoforms arise via the use of alternative transcription start sites within the *RGS6* gene sequence. Specially, while RGS6L protein isoforms are generated using a transcription start site upstream of exon 1 of the *RGS6* gene, RGS6S isoforms arise via a transcription start site within intron 7. Therefore, RGS6S protein isoforms lack the first 140 amino acids present in the N-terminus of RGS6L isoforms, and therefore also lack the DEP/DHEX domain (Fig. 3*A*). The DEP/DHEX domain is known to promote membrane targeting of R7 family members by facilitating their interaction with two membrane proteins, the retina-specific RGS9 anchor protein (R9AP) and its close relative, R7 family binding protein (R7BP) which is ubiquitously expressed (Fig. 3*A*; Lishko et al., 2002; Hu and Wensel, 2002; Martemyanov et al., 2003, 2005; Drenan et al., 2005; Anderson et al., 2007; Patil et al., 2018). However, the DEP/DHEX domain may also facilitate the interaction between Gβ_5_ and R7 family members (Cheever et al., 2008). Recent evidence supports a model whereby R9AP/R7BP strengthens the interaction between the DEP/DHEX and GGL domains with Gβ_5_ promoting R7 RGS protein stabilization (Masuho et al., 2011; Patil et al., 2018). These findings are consistent with our cycloheximide chase experiments demonstrating that RGS6L isoforms are more stable than RGS6S isoforms (Fig. 4*D*).

### Identification of novel RGS6 protein bands

We identified three RGS6 protein bands that were larger (61-, 65-, and 69-kDa) than any protein isoform predicted from our initial cloning effort (≤57-kDa, Chatterjee et al., 2003). All three bands were RGS6L isoforms as they were recognized by the RGS6-L antibody (Figs. 4*A*, 5). Of these, the 65- and 69-kDa bands appear to be brain-specific (Fig. 5) phospho (p-RGS6B) and dephospho (RGS6B) forms of RGS6L, respectively. The 65-kDa p-RGS6B band, whose expression is reduced in the presence of λ phosphatase and increased by a Ca^2+^-stimulated kinase activity, represents the first positively identified RGS6 phospho-protein (Fig. 7*B–E*). In contrast, the 69-kDa RGS6B protein band is increased by λ phosphatase (Fig. 7*B*) and decreased by Ca^2+-^stimulated kinase activity (Fig. 7*E*). Both the 61- and 69-kDa RGS6 isoforms are much larger than predicted from any transcripts identified in our original cloning effort (Chatterjee et al., 2003), suggesting they may represent novel RGS6 isoforms. In support of this hypothesis, northern blot analyses conducted in human brain and peripheral tissues indicate that there is at least one transcript predominantly expressed in the CNS that is larger than the other RGS6 transcripts (which correspond in size to the RGS6L and RGS6S splice forms; Snow et al., 1999).

Of the novel RGS6 protein bands described above, the 69-kDa RGS6B isoform is of particular interest. Importantly, we have shown that RGS6-specific inhibition of Gα_i/o_ modulates several CNS disorders for which RGS6 may be a novel therapeutic target. Remarkably, RGS6^−/−^ mice have reduced anxiety/depression (Stewart et al., 2014), exhibit diminished alcohol seeking/reward (Stewart et al., 2015), and develop Parkinson’s disease (Bifsha et al., 2014; Luo et al., 2019; Fig. 1). Re-examination of the Western blot data presented in these papers revealed that RGS6B is highly expressed in the brain regions affected by the CNS disorders described above, suggesting it plays a critical role in CNS pathology. Therefore, future studies should focus on identifying the transcript encoding RGS6B so that its function may be further explored. There are several pieces of evidence derived from this study that may aid in transcript identification. First, RGS6B is recognized by the RGS6-L antibody (Fig. 4*A*, middle panel, green arrow), indicating the transcript will contain exons 1–6, which encode the long N-terminus. Second, RGS6B is also recognized by the RGS6-18 antibody (Fig. 4*A*, right panel, green arrow), indicating that the transcript includes exon 18. Finally, RGS6B is present in both mouse and human (Fig. 8*B*), indicating it is highly conserved. Therefore, the sequence of the identified transcript must be derived from highly conserved regions within the *RGS6* gene.

### Speculating on the possible effects of the rs2332700 SNP on RGS6 expression

In reflecting on the present data and the recent meta-analysis (Cross-Disorder Group of the Psychiatric Genomics Consortium, 2019) which linked a SNP (rs2332700) in *RGS6* to autism spectrum disorder, bipolar disorder, major depression, and schizophrenia, it becomes intriguing to speculate on the SNP’s functionality. The rs2332700 SNP maps to intron 1 of the *RGS6* gene and is associated with a G > C switch. Interestingly, this SNP is only found in primates (Extended Data Fig. 5-2). While a functional role of this SNP has yet to be established, we hypothesize that it alters RGS6 expression. As RGS6L(+GGL) protein isoforms predominate (Fig. 5*A*, magenta arrow), we hypothesize that it is the physiological functions of these isoforms most highly impacted by the SNP. Interestingly, the RGS6L(+GGL) isoforms predominate not only in the CNS, but also in the periphery (Fig. 5), suggesting that the rs2332700 SNP may not only be linked to the psychological pathology of schizophrenia, autism spectrum disorder, bipolar disorder, and major depression, but also peripheral comorbidities associated with these diseases. In support of this hypothesis, we have demonstrated that global loss of RGS6 not only reduces CNS-associated alcohol seeking and reward behavior, in mice, but also protects against peripheral alcohol-mediated heart, liver, and gastrointestinal tract toxicity (Stewart et al., 2015).

Another intriguing piece of data from our current study is the discovery of a 69-kDa form of RGS6 that is brain-specific (RGS6B). Although the functional nature of RGS6B is unknown, numerous Western blot analyses conducted in this paper and others (Bifsha et al., 2014; Stewart et al., 2014, 2015; Luo et al., 2019) demonstrate that it is highly expressed in several brain regions associated with numerous psychiatric disorders including, but not limited to, alcohol use disorders, anxiety/depression, and Parkinson’s disease. In addition, our current data suggest that RGS6B shares several structurally similarities with the other predominant RGS6L(+GGL) isoforms (Fig. 4*A*). Therefore, it is intriguing to speculate that RGS6B expression may also be altered by the rs2332700 SNP. However, as RGS6B is not expressed in the periphery, we may surmise that alteration in its functional role would largely have psychological effects.

## Synthesis

Reviewing Editor: Michael Michaelides, NIDA-NIH Decisions are customarily a result of the Reviewing Editor and the peer reviewers coming together and discussing their recommendations until a consensus is reached. When revisions are invited, a fact-based synthesis statement explaining their decision and outlining what is needed to prepare a revision will be listed below. The following reviewer(s) agreed to reveal their identity: Venetia Zachariou, Benita Sjogren. Note: If this manuscript was transferred from JNeurosci and a decision was made to accept the manuscript without peer review, a brief statement to this effect will instead be what is listed below.

Reviewer 1

The study by Ahlers-Dannen and colleagues focuses on the characterization and tissue expression profiling of RGS6 protein isoforms. RGS6 is expressed in peripheral and central tissues, whereas several RGS6 isoforms have been implicated in neuropsychiatric disorders. A better understanding of the function and regulation of each form may provide critical insight into disease pathophysiology and treatment possibilities. RGS6 variants may contain longer or shorter N terminal domains or they may have alternative C termini, whereas variants with incomplete GGL domains, have also been identified. These structural differences suggest that specific variants may have unique function and cellular/regional localization. In this study, the investigators utilized multiple methodologies and three novel antibodies they have developed in order to characterize new forms of RGS6. Interestingly, the investigators identified a 69kd phosphoprotein form expressed in the brain (in rodents and humans), which is expected to play critical roles in CNS disorders and physiological function.

This is a well written paper, that enhances our understanding of RGS protein function and their implication in human disorders. The new antibodies permit the study of a brain specific phosphoprotein which may play unique roles in neuropsychiatric disorders. I do not have any edits or additional suggestions, as the manuscript is clearly written and the data are clearly presented.

Reviewer 2

The manuscript entitled “Protein profiling of RGS6, a pleiotropic gene implicated in numerous neuropsychiatric disorders, reveals multi-isoformic expression and a novel brain-specific isoform” describes the development of isoform-specific antibodies against Regulator of G protein Signaling 6 (RGS6), a protein that has been implicated in several disorders associated with the CNS. They also identify a novel, brain specific isoform, which could have implications in these disorders. This manuscript is interesting and timely, particularly given the increasing recognition of RGS proteins as drug targets and key regulators of cellular signaling. In addition, given that the majority of available RGS protein antibodies are of poor quality, the development of these antibodies will be a helpful tool in the study of RGS6.

Below I have outlined some points that, if addressed, would enhance the impact of the presented results:

1) Regarding the determination of the 65kDa as a phosphorylated isoform: These data are not convincing. Why would the band simply disappear upon treatment with λ phosphatase? Is the dephosphorylated protein that unstable? An additional measure of protein phosphorylation should be attempted, such as demonstrating mobility shift (e.g. PhosTag). In Fig.7 the authors present a multitude of potential phosphorylation sites. Why would only one isoform be phosphorylated? These studies need further clarification and additional explanation. The authors conclusion that the “69kDa RGS6 protein band is not a phosphor-protein” on page 17 (line 417) is not convincing at this point.

2) Do the RGS6 isoforms lacking a complete GGL domain still bind Gβ5? If not, that would be a clear reason for their instability. Adding these data or at least adding a discussion would help the interpretation of results.

3) The results in Fig. 9B are troublesome:

a. I assume that the first lane is a control of some sort, but nowhere is it mentioned what was used.

b. RGS6 was not shown in this figure.

c. αTub was used as a loading control, but it is not detected in all lanes, making this reviewer question the equal loading of protein in each lane. If αTub could not be used, the authors should include a different loading control.

d. The authors also do not mention the identity of the RGS7, 9 and 11 antibodies that were used. Given the questionable quality of many RGS protein antibodies, this is an important piece of information.

e. In particular, the RGS9 ‘control’ lane does not convince me that this antibody works. Also, it is labeled ‘RGS9-1’, which to my knowledge is the isoform only expressed in the retina...

4) The authors claim to identified a novel RGS6 isoform, yet on page 24 (lines 563-564) they refer to western blot analyses by them and others dating back to 2014. In checking one of their own references (Luo et al. 2019), they did not blot for RGS6 in that paper. The text should be changed and clarified. This point raises the issue that there may be more instances of inappropriate referencing throughout the manuscript. The authors should carefully go through the text and cross check.

5) In the methods section, the authors mention a construct for R7BP (gift from K. Martemyanov) used for transfection but there is no mention of it in the results. It appears it was not used in this study?

The manuscript was made more difficult to review because the figures were not numbered in the manuscript file. This may have been an artifact during submission but it made it harder to follow.
