# Supplementary figures and images for: Protein Profiling of RGS6, a Pleiotropic Gene Implicated in Numerous Neuropsychiatric Disorders, Reveals Multi-Isoformic Expression and a Novel Brain-Specific Isoform
Source: eNeuro. 2022 Jan 18;9(1):ENEURO.0379-21.2021. doi: 10.1523/ENEURO.0379-21.2021 (PMC8805202; doi:10.1523/ENEURO.0379-21.2021)

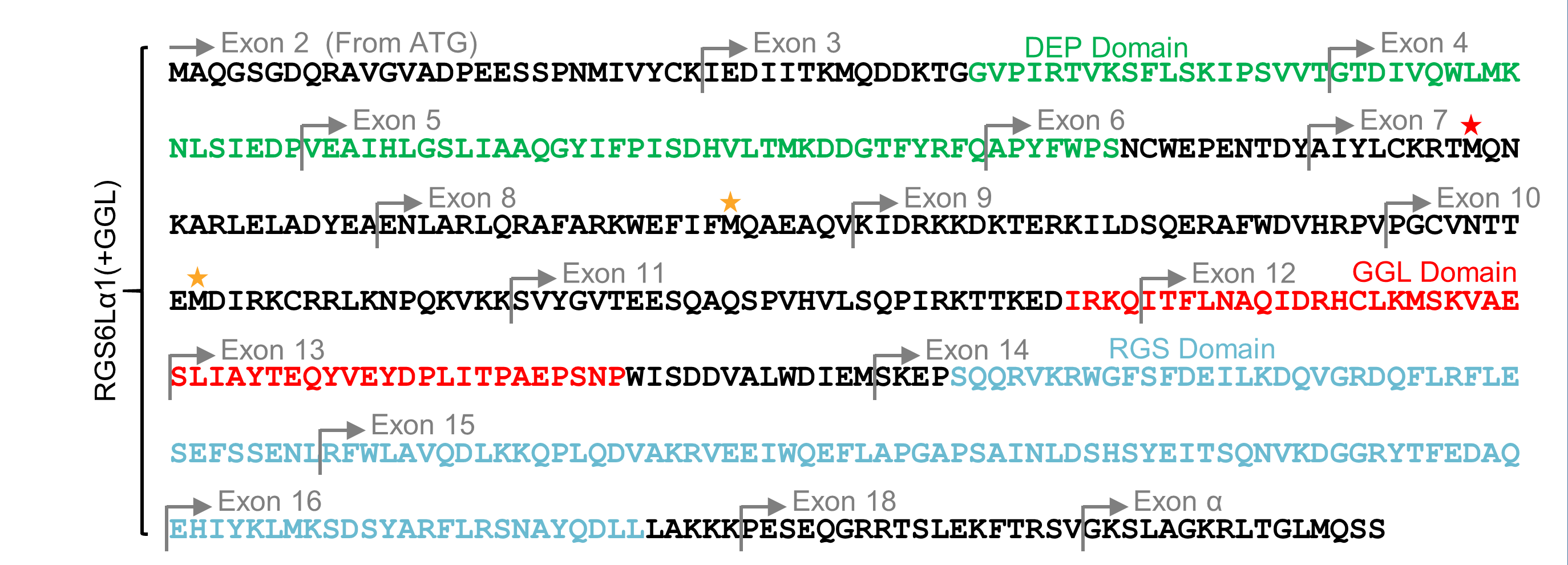

Supplement: Extended Data Figure 4-2 — Novel putative translation start sites identified within RGS6 protein sequence. Predicted RGS6Lα1 protein sequence. Red star denotes alternative start site utilized to produce RGS6S isoforms. Yellow stars denote novel putative translation start sites present in both RGS6L and RGS6S isoforms. Download Figure 4-2, TIF file. [file enu-eN-NWR-0379-21-s03.tif]

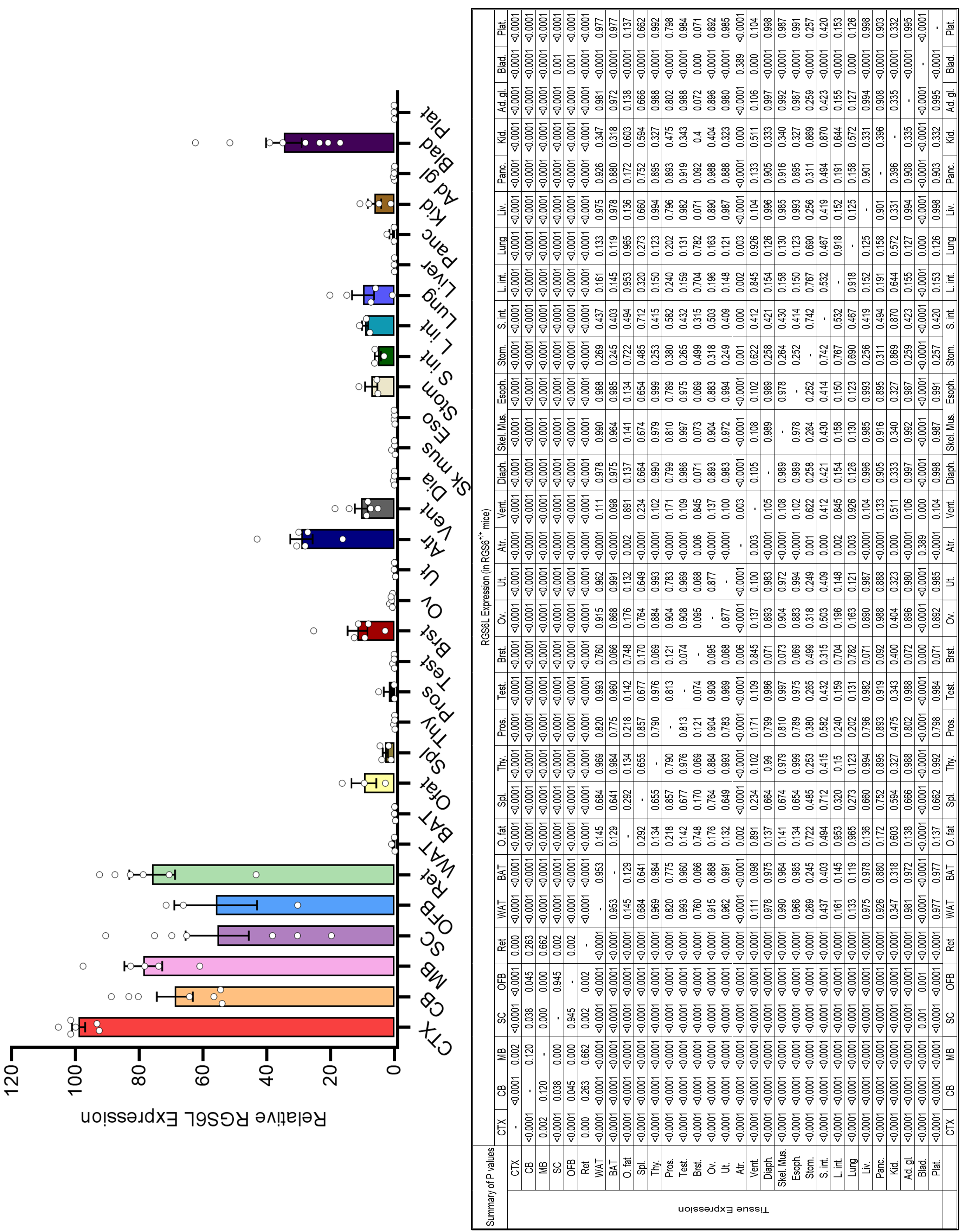

Supplement: Extended Data Figure 5-1 — Comparison of RGS6 expression across 31 mouse tissues using the RGS6-L antibody. Using the RGS6+/+ (WT) tissue data from Figure 5, we have graphed RGS6 protein expression detected with the RGS6-L antibody across the same 31 tissues. Here, we find that RGS6 is most highly expressed in the cerebral cortex (CTX, p ≤ 0.000) relative to all other tissues tested. Data are represented as mean ± SEM. Data were analyzed via one-way ANOVA with Fisher’s LSD post hoc adjustment. Table below displays the p values of all comparisons of RGS6 expression across the various tissues. CTX = cerebral cortex, OFB = olfactory bulb, MB = midbrain, CB = cerebellum, SC = spinal cord, Ret = retina, WAT = white adipose tissue, BAT = brown adipose tissue, Ofat = Omental fat, Spl = spleen, Thy = thymus, Pros = prostate, Test = testis, Brst = breast, Ov = ovary, Ut = uterus, Atr = atrium, Vent = ventricle, Dia = diaphragm, Sk mus = skeletal muscle, Eso = esophagus, Stom = stomach, S int = small intestine, L int = large intestine, Panc = pancreas, Kid = kidney, Ad gl = adrenal gland, Blad = bladder, Plat = platelet. Download Figure 5-1, TIF file. [file enu-eN-NWR-0379-21-s04.tif]

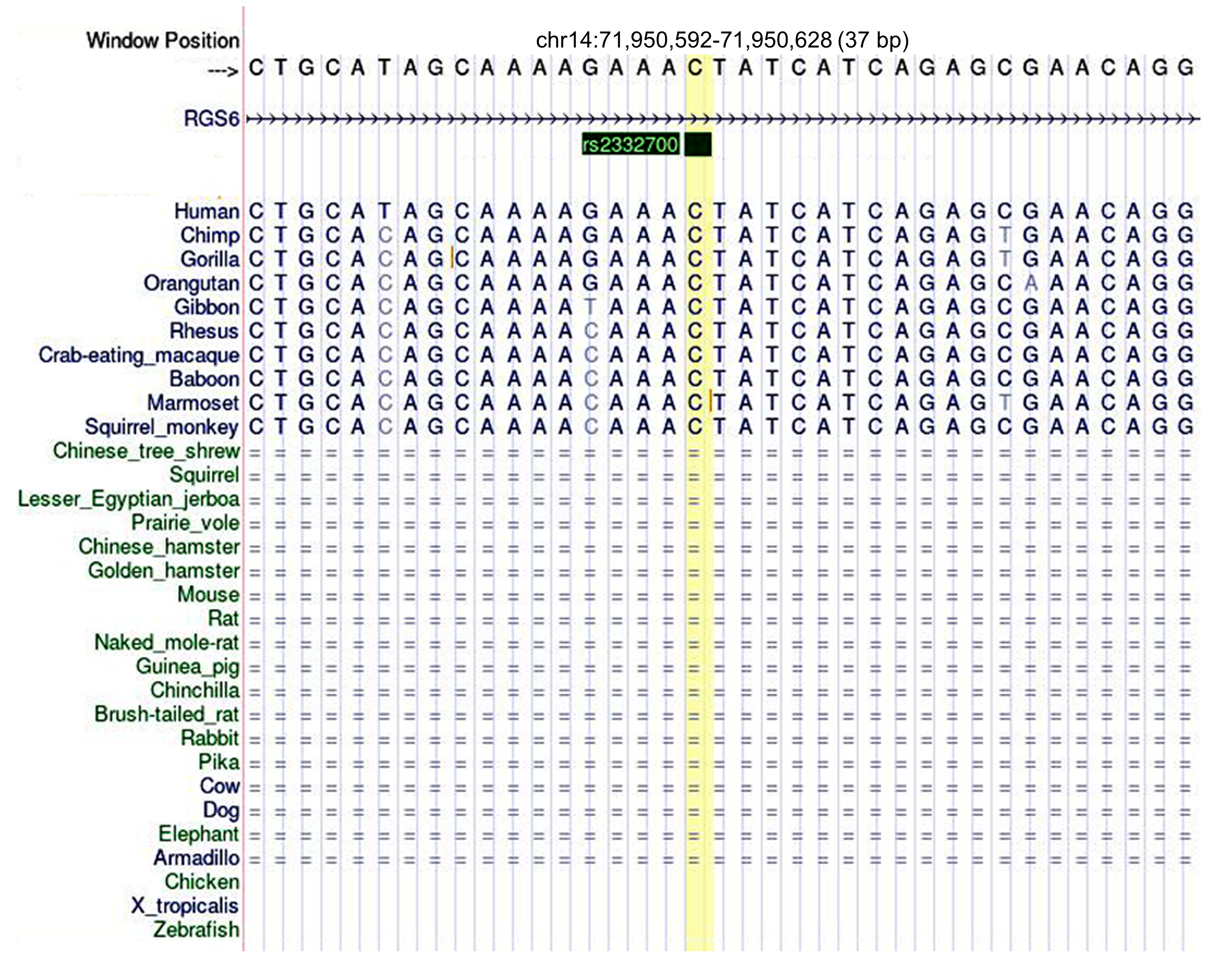

Supplement: Extended Data Figure 5-2 — Evolutionary conservation of the rs2332700 SNP. Human RGS6 sequence (37 bps) flanking the rs2332700 SNP (G > C) aligned against the corresponding sequence present in 30 different animal species across the animal kingdom using the UCSC Genome Browser. The SNP is only found in primates. =: gap regions in genomes, in which aligning species has one or more unalignable bases. This is likely due to excessive evolutionary distance between the aligning species and human or due to independent insertions/deletions in the region between the aligning species and human. Download Figure 5-2, TIF file. [file enu-eN-NWR-0379-21-s05.tif]

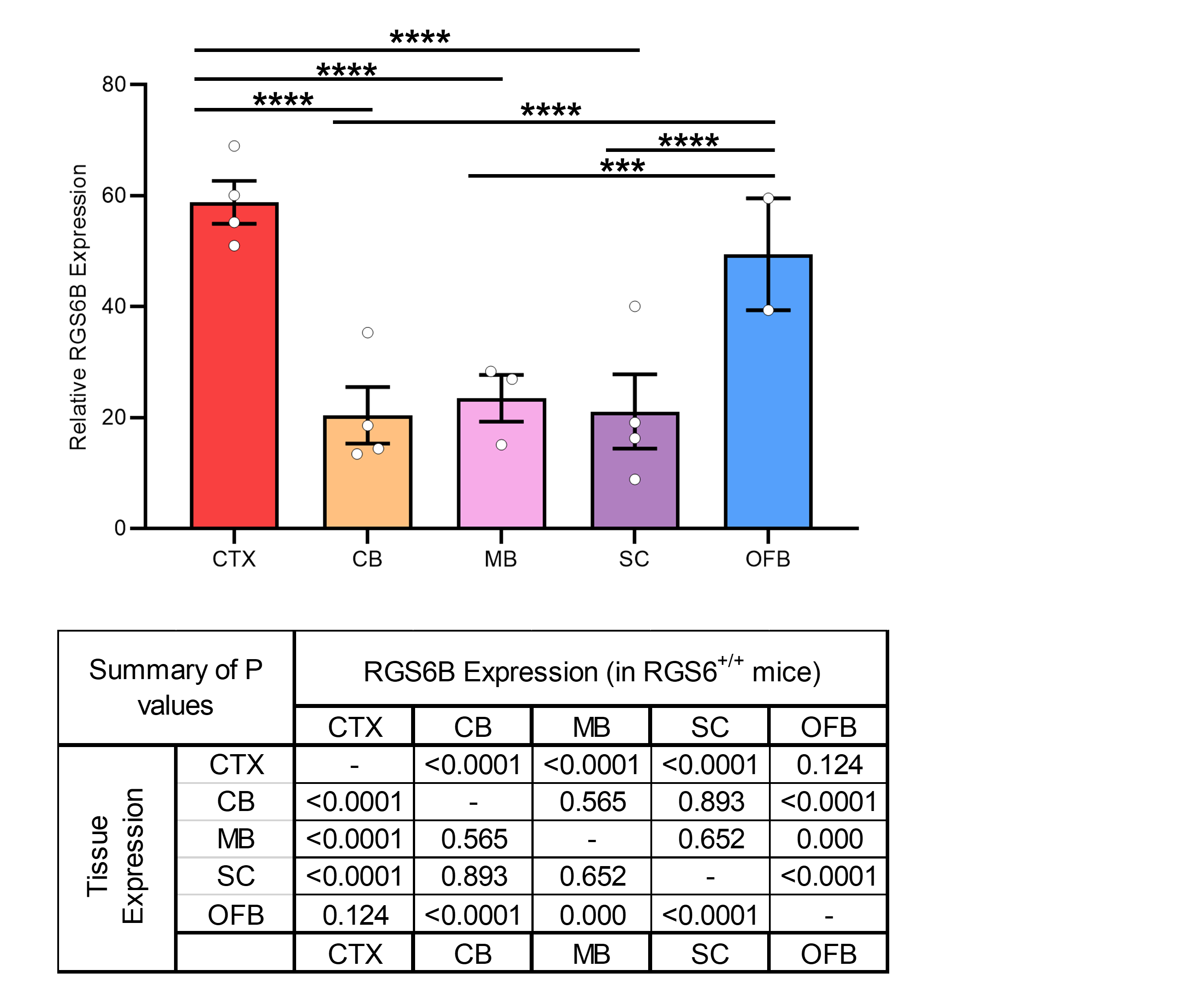

Supplement: Extended Data Figure 8-1 — Comparison of RGS6B expression across CNS mouse tissues using the RGS6-L antibody. Using the RGS6+/+ (WT) tissue data from Figure 8, we have graphed RGS6B protein expression detected with the RGS6-L antibody across mouse CNS tissues. Data are represented as mean ± SEM. Data were analyzed via one-way ANOVA with Fisher’s LSD post hoc adjustment. Table below displays the p values of all comparisons of RGS6 expression across the various tissues. CTX = cerebral cortex, CB = cerebellum, MB = midbrain, SC = spinal cord, OFB = olfactory bulb. Download Figure 8-1, TIF file. [file enu-eN-NWR-0379-21-s07.tif]

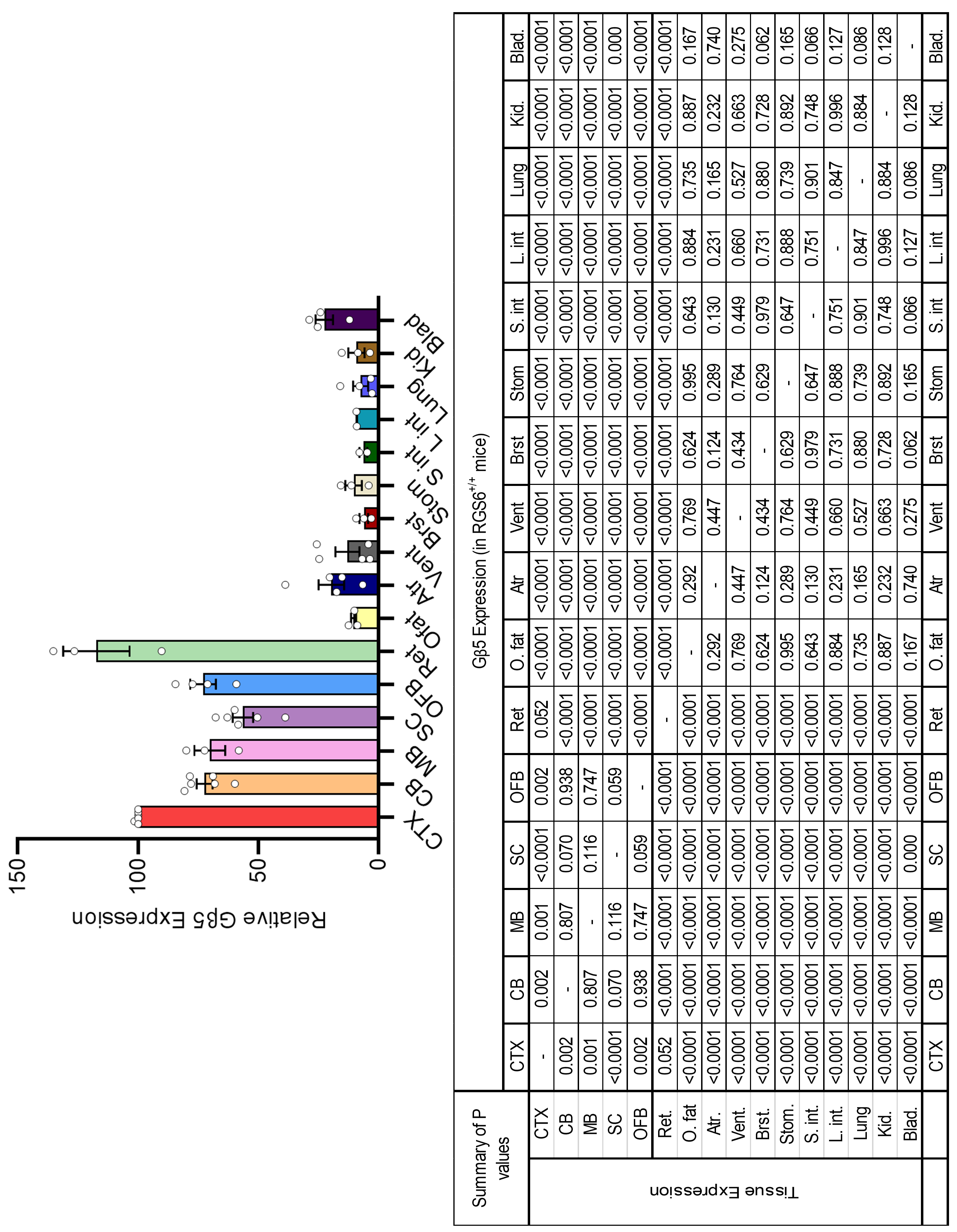

Supplement: Extended Data Figure 9-1 — Comparison of Gβ5 expression across 16 mouse tissues. Using the RGS6+/+ (WT) tissue data from Figure 9, we have graphed Gβ5 protein expression across 16 mouse tissues. Data are represented as mean ± SEM. Data were analyzed via one-way ANOVA with Fisher’s LSD post hoc adjustment. Table below displays the p values of all comparisons of RGS6 expression across the various tissues. CTX = cerebral cortex, CB = cerebellum, MB = midbrain, SC = spinal cord, OFB = olfactory bulb, Ret = retina, Ofat = Omental fat, Atr = atrium, Vent = ventricle, Brst = breast, Stom = stomach, S int = small intestine, L int = large intestine, Kid = kidney, Blad = bladder. Download Figure 9-1, TIF file. [file enu-eN-NWR-0379-21-s08.tif]
